# Regulation of Gene Expression Patterns in Mosquito Reproduction

**DOI:** 10.1371/journal.pgen.1005450

**Published:** 2015-08-14

**Authors:** Sourav Roy, Tusar T. Saha, Lisa Johnson, Bo Zhao, Jisu Ha, Kevin P. White, Thomas Girke, Zhen Zou, Alexander S. Raikhel

**Affiliations:** 1 Department of Entomology, University of California, Riverside, Riverside, California, United States of America; 2 Institute of Integrative Genome Biology, University of California, Riverside, Riverside, California, United States of America; 3 Graduate Program in Cell, Molecular and Developmental Biology, University of California, Riverside, Riverside, California, United States of America; 4 Graduate Program in Genetics, Genomics and Bioinformatics, University of California, Riverside, Riverside, California, United States of America; 5 Institute for Genomics and Systems Biology, University of Chicago, Chicago, Illinois, United States of America; 6 Department of Botany and Plant Sciences, University of California, Riverside, Riverside, California, United States of America; 7 State Key Laboratory of Integrated Management of Pest Insects and Rodents, Institute of Zoology, Chinese Academy of Sciences, Beijing, China; Howard Hughes Medical Institute, UNITED STATES

## Abstract

In multicellular organisms, development, growth and reproduction require coordinated expression of numerous functional and regulatory genes. Insects, in addition to being the most speciose animal group with enormous biological and economical significance, represent outstanding model organisms for studying regulation of synchronized gene expression due to their rapid development and reproduction. Disease-transmitting female mosquitoes have adapted uniquely for ingestion and utilization of the huge blood meal required for swift reproductive events to complete egg development within a 72-h period. We investigated the network of regulatory factors mediating sequential gene expression in the fat body, a multifunctional organ analogous to the vertebrate liver and adipose tissue, of the female *Aedes aegypti* mosquito. Transcriptomic and bioinformatics analyses revealed that ~7500 transcripts are differentially expressed in four sequential waves during the 72-h reproductive period. A combination of RNA-interference gene-silencing and *in-vitro* organ culture identified the major regulators for each of these waves. Amino acids (AAs) regulate the first wave of gene activation between 3 h and 12 h post-blood meal (PBM). During the second wave, between 12 h and 36 h, most genes are highly upregulated by a synergistic action of AAs, 20-hydroxyecdysone (20E) and the Ecdysone-Receptor (EcR). Between 36 h and 48 h, the third wave of gene activation—regulated mainly by HR3—occurs. Juvenile Hormone (JH) and its receptor Methoprene-Tolerant (Met) are major regulators for the final wave between 48 h and 72 h. Each of these key regulators also has repressive effects on one or more gene sets. Our study provides a better understanding of the complexity of the regulatory mechanisms related to temporal coordination of gene expression during reproduction. We have detected the novel function of 20E/EcR responsible for transcriptional repression. This study also reveals the previously unidentified large-scale effects of HR3 and JH/Met on transcriptional regulation during the termination of vitellogenesis and remodeling of the fat body.

## Author Summary

In addition to being vectors of devastating human diseases, mosquitoes represent outstanding model organisms for studying regulatory mechanisms of differential gene expression due to their rapid reproductive cycles. About 7500 transcripts are differentially expressed in four sequential waves during the 72-h reproductive period in the fat body, a critical reproductive organ. The major regulators for these waves of gene expression are the two very important insect hormones, 20-hydroxyecdysone (20E) and Juvenile hormone (JH), their respective receptors Ecdysone Receptor (EcR) and Methoprene-Tolerant (Met), amino acids and the orphan nuclear receptor HR3. These key regulators are responsible for activation and repression of co-regulated gene sets, at different time points, within the 72-h reproductive period. Importantly, this study, apart from providing an insight into the regulatory complexity involved in the temporal coordination of gene expression, also reveals the previously unidentified roles of 20E/EcR, JH/Met and HR3 during the 72-h period post blood meal.

## Introduction

Numerous studies in model organisms have identified patterns of gene expression correlated with embryogenesis and development [1–9]. These studies have eloquently demonstrated the existence of a tight coordination between large gene cohorts and various stages of a developing organism on a spatiotemporal scale. In contrast, investigation of genomic profiles during reproduction has attracted much less attention. Blood-feeding animals such as mosquitoes, in addition to being vectors of numerous devastating human diseases, represent outstanding models because their reproductive events are synchronized by the intake of blood and occur within a short time span. Moreover, their reproduction is cyclic, with each cycle of egg development linked to a separate blood-feeding event. Previous studies have identified differential gene expression associated with blood feeding in the malaria mosquito *Anopheles gambiae* and the Dengue virus vector mosquito *Aedes aegypti* [10–12]. However, temporal control of gene expression patterns during blood-meal-activated mosquito reproduction is not yet completely understood.

The gonadotrophic cycle of a female mosquito is divided into two periods: pre- and post-blood meal. In the *A*. *aegypti* female, the pre-blood meal period, which in the first gonadotrophic cycle also includes post-eclosion (PE) development, lasts from at least 72 h until the mosquito takes a blood meal. It is controlled by juvenile hormone (JH) and its receptor Methoprene-tolerant (Met) [13, 14]. Both amino acid/Target of Rapamycin nutritional signaling and insulin are essential for activating post-blood-meal (PBM) events in the gut, ovaries and the fat body [15–19]. 20-Hydroxyecdysone (20E) is the main regulator of PBM events in the fat body, which produces yolk protein precursors (YPPs) for subsequent egg development [13, 20]. In the *A*. *aegypti* female, it takes 72 h to complete the entire PBM period.

During each gonadotrophic cycle, the fat body undergoes dramatic changes, shifting its functions from acting as a storage depot for lipid and carbohydrate reserves to becoming an immense protein-producing factory [20)]. At the end of the gonadotrophic cycle, it undergoes programmed autophagy and transforms itself back to reserve storage [21]. Hence, this tissue is particularly useful for studies of temporal coordination of gene expression. Our previous study [14] revealed gene expression patterns in the fat body during the pre-blood-meal period of the first gonadotrophic cycle in the *A*. *aegypti* female mosquitoes. We have shown that while metabolic genes are expressed early, those encoding transcription and translation machineries get activated later during this period. Moreover, we demonstrated that while the former group of genes is repressed by JH and Met, the latter is activated by these factors [14].

Here, we investigated the network of regulatory factors responsible for sequential gene expression in the PBM fat body. We show that systemic factors—JH, 20E and nutritional amino acids (AAs)—differentially regulate this gene-expression program. Moreover, our study has revealed that JH and 20E signaling in the PBM fat body is mediated by Met, EcR and HR3. Importantly, we report the previously unidentified role of JH in controlling gene expression during the PBM period. Furthermore, we have demonstrated the repressive function of 20E, which downregulates large cohorts of PBM genes in this mosquito tissue. Finally, we have shown that EcR mediates this repressive function. Taken together, our study provides new insights into the complexity of regulatory mechanisms responsible for temporal coordination of gene expression during reproduction in the female *A*. *aegypti* mosquito.

## Results

### Differential gene expression in the blood-meal-activated fat body of the female *Aedes aegypti*

**Fig 1:**
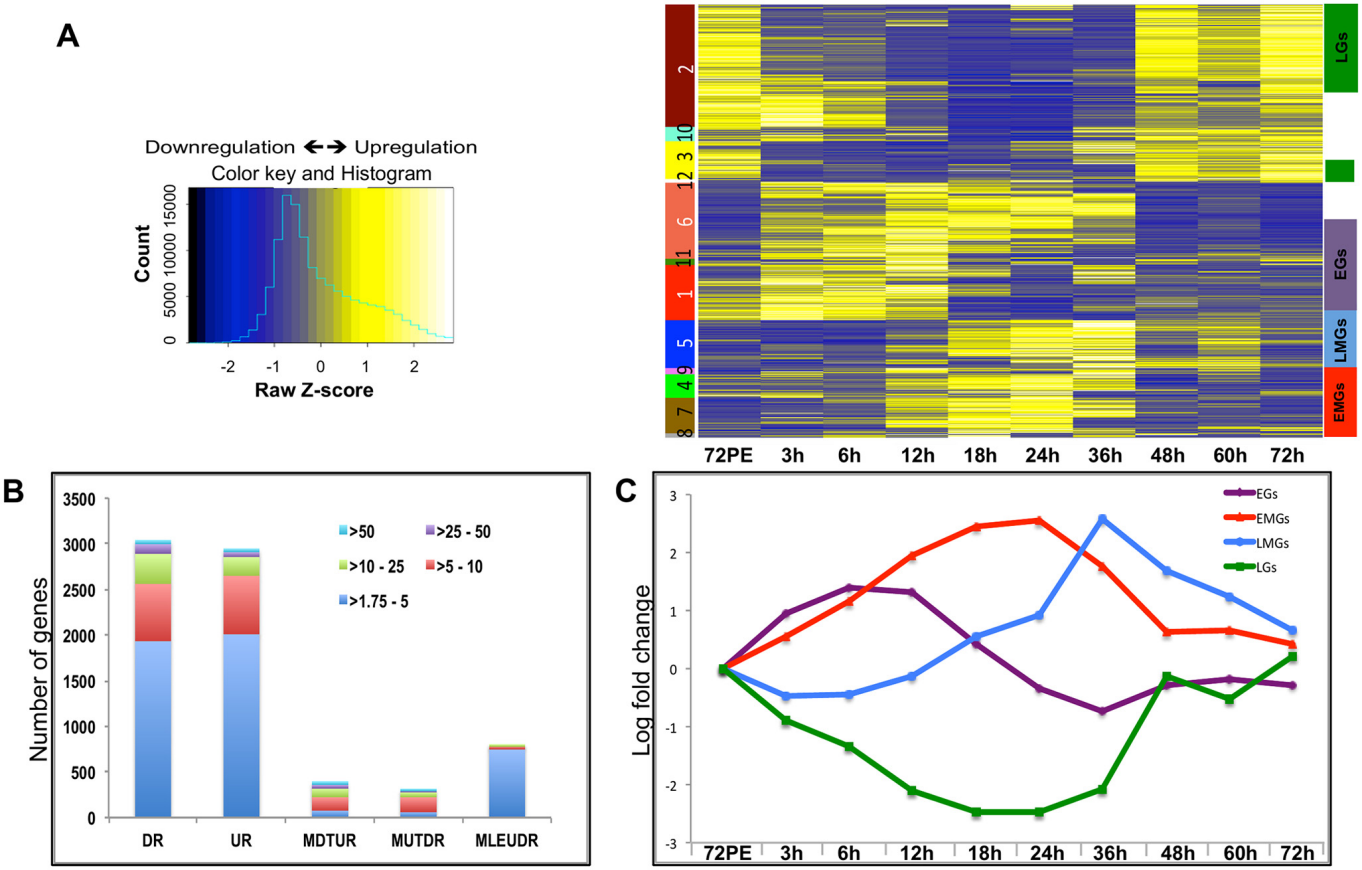
Expression dynamics of *A*. *aegypti* fat body genes post blood meal. (**A**) The heatmap shows the expression values (mean among three replicates) at nine different time points. Each row represents data from one transcript, and each column corresponds to a time point between 3 h and 72 h PBM. Hierarchical clustering with subsequent tree cutting identified 12 discrete clusters, which are color coded on the left. Clusters 1, 11 and 6 were grouped as EGs, clusters 8, 7 and 4 as the EMGs, cluster 5 carried the LMGs and clusters 2 and 3 were grouped as LGs. The color legend of the row-wise normalized expression values (Z-scores) is given on the left. **(B)** The stacked columns show the breakdown of the DEGs provided in Fig 1A and 1B –source data. The different colors represent the fold changes e.g. red signifies number of genes that were differentially expressed by >5 fold. DR—Down Regulated, UR—Up Regulated, MDTUR—More Down Than Up Regulated, MUTDR—More UP Than Downregulated, MLEUDR—More or Less Equally UP and Down Regulated. **(C)** Average expression profiles of all EGs (purple line), EMGs (red line), LMGs (blue line) and LGs (green line) shown, X- axis shows the different time points while the Y-axis displays the average log fold change (See S2 Dataset).

The goal of this study was to obtain detailed information about differential gene expression dynamics and to elucidate the regulatory networks governing the complex gene expression patterns following blood meal activation of reproductive events in female mosquitoes. We addressed these issues using the fat body, because it constitutes a tissue critical for female reproduction and is highly amenable for experimental studies in this organism, which lacks well-established genetics [20]. Custom-made Agilent microarray chips containing probe sets corresponding to 15,321 *A*. *aegypti* genes [14, 22] were used to examine the fat-body tissue samples collected at nine time points, spanning from 3 h to 72 h PBM. Differentially expressed gene (DEG) sets were established by comparing transcripts from each of the nine time points with that from the fat body of pre-blood-meal female mosquitoes, 72 h PE, using a minimum fold change of ≥1.75 (0.8 in a log2 scale) as the confidence threshold and a false-discovery rate (adjusted *P* value) of ≤0.01, similar to the criteria used by Zou et al., 2011, 2013. When applying this threshold, 7468 transcripts, which constituted almost half of the total number of genes probed in the *A*. *aegypti* genome, displayed differential expression during at least one of the nine time points within the 72-h period PBM, in the fat body of female *Aedes* mosquitoes (Fig 1A and see S1 Dataset). 3045 transcripts were found to be downregulated, 2938 upregulated and 1485 were found to be both up- and downregulated during different time points within the 72-h period PBM (Fig 1B and see S1 Dataset). Of these 1485 transcripts, 395 were downregulated to a greater extent than were upregulated (a difference of >1.75 between the fold changes), 306 were more upregulated than downregulated applying the same criterion (Fig 1B and see S1 Dataset), and 784 were almost equally up- and downregulated at different time points (a difference of <1.75 between the fold changes). More than 2500 transcripts displayed differential expression of greater than 5-fold (Fig 1B and see S1 Dataset).

Unlike during the PE period, when the number of DEGs increased consistently to reach a maximum during late PE (60–66 h) [14], the number of DEGs during the PBM period started to increase at around 12 h, reached a maximum between 18 h and 24 h, and then decreased sharply after 36 h PBM (S1 Fig).

Hierarchical clustering of the DEGs resulted in 12 different clusters (Fig 1A). Genes within certain clusters displayed similar expression profiles, barring minor variations. As a result, most of the genes could be categorized into four broad sets, depending on their expression profiles and the time of their maximal expression: early genes (EGs), early-mid genes (EMGs), late-mid genes (LMGs) and late genes (LGs) (Fig 1A). Transcript levels of EGs (Clusters 1, 11 and 6) were elevated by 3 h PBM, reached their maximum levels between 6 h and 12 h PBM, and declined between 18 h and 36 h, before getting slightly elevated again between 48 h and 72 h PBM (Fig 1C and see S2 Dataset). In comparison, EMG transcript levels (Clusters 8, 7, 4) did not show significant increase until 12 h PBM, reached the maximum levels between 18 h and 24 h, after which their expression declined by 36 h PBM (Fig 1C and see S2 Dataset). The genes that displayed a low expression prior to 24 h PBM followed by a sudden increase within 36–48 h PBM and a sharp decline post 48 h were grouped as the LMGs (Cluster 5; Fig 1C and see S2 Dataset). The LGs (Clusters 2 and 3) are those showing a decline in their expression following a blood meal, maintaining low expression during early-mid and late-mid periods, and showing maximum expression between 48 h and 72 h (Fig 1C and see S2 Dataset). Expression patterns of fat body genes were confirmed by means of quantitative real-time polymerase chain reaction (qRT-PCR) analysis; the profiles obtained by measuring the transcript levels of selected EGs, EMGs, LMGs and LGs, using qRT-PCR, displayed good correlation with the microarray data (S2 Fig).

Overall, our microarray analysis revealed an extremely high level of transcriptional activity in the fat body during the PBM period of the gonadotrophic cycle. Moreover, we were able to identify four major sequential waves of gene expression over the 72-h period PBM in the female fat body.

### Temporal separation of functional gene groups expressed during the PBM period

**Fig 2:**
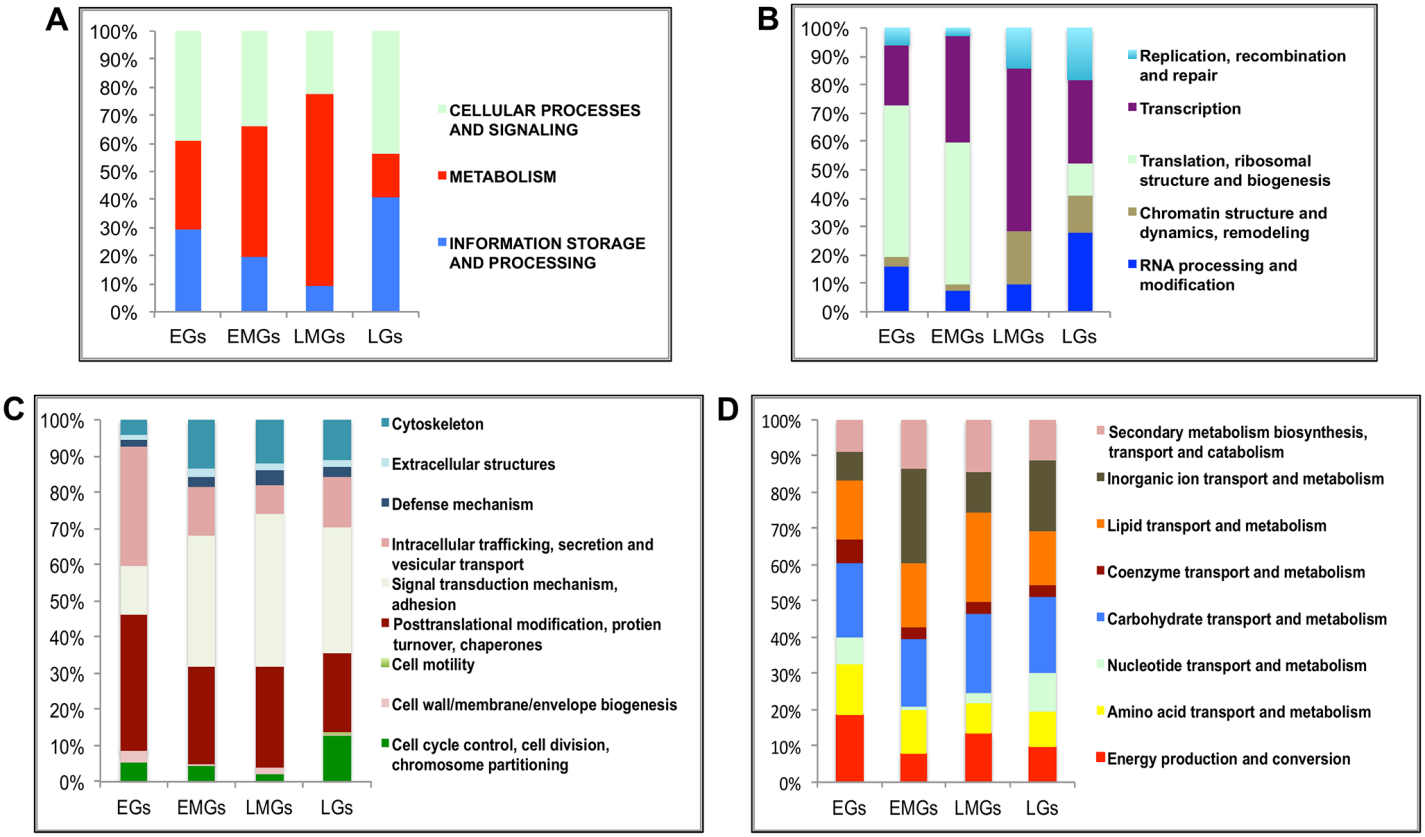
Functional group enrichment analysis of EGs, EMGs, LMGs and LGs. **(A)** Analyses of functional groups within the EG, EMG, LMG and LG sets using the inNOG database. The Y-axis of this 100% stacked columns show the percentage of genes that fall within each functional group (see Fig 2—source data). **(B-D)** A more detailed analysis of the same gene cohorts using the inNOG database (see Fig 2—source data). **(B)** The enrichment of IS&P genes within more specific functional categories determined. **(C)** The enrichment of CP&S genes within more specific functional categories determined. **(D)** The enrichment of metabolism genes within more specific functional categories determined.

To understand the functional identity of genes expressed during the PBM period in the fat body, the EGs, EMGs, LMGs and LGs were examined by searching the inNOG (Insects non-supervised orthologous groups and their proteins) within the eggNOG v3.0 (evolutionary genealogy of genes: Non-supervised Orthologous Groups) database [23]. This analysis revealed that multiple changes occur in the functional identity of the fat body transcriptome over the duration of 72 h PBM. We observed that 40% of the EGs belong to cellular processes and signaling (CP&S) gene category, 30% to metabolism (MT) and 30% to information storage and processing (IS&P) (Fig 2A and see S3 Dataset). In the case of the EMGs, the percentage of MT genes increases to ~45%, while the percentages of the other two groups decreases (~35% CP&S; ~20% IS&P) (Fig 2A). This trend continues for the LMGs, for which almost 70% are MT genes, with about 10% IS&P genes and about 20% CP&S genes (Fig 2A). However, this trend completely reverses with the LGs, for which only about 15% of the total genes is represented by the MT genes, and both CP&S and IS&P constitute a little over 40% each (Fig 2A).

It was observed that while most MT genes are active during the early-mid and late-mid PBM periods, there is an enrichment of CP&S and IS&P during the early and late PBM periods. To examine the functional dynamics on a finer scale, we further sorted these genes into more-specific functional categories within the inNOG database (Fig 2B–2D and see S3 Dataset). The results of this analysis revealed a remarkable temporal separation of major functional gene categories over the 72 h PBM. Separation of IS&P genes into finer functional categories revealed that more than 70% of EGs are transcription (TR) and translation, ribosome structure and biogenesis (TRB) genes, where TRB alone constitutes more than 50% of the genes (Fig 2B). The percentage of TR genes increases in EMGs (>35%) and further in LMGs (55%), whereas, the TRB genes show an opposite trend, these genes seem to be significantly downregulated during the late-mid and late stages (Fig 2B).

Similarly, a closer look at the CP&S groups showed that while signal transduction mechanism (STM) genes constitute a little over 10% of CP&S amongst EGs, the percentage increases to ~45% in LMGs and decreases slightly in LGs (Fig 2C). Conversely, intracellular trafficking, secretion and vesicular transport (ITS&VT) show an exact opposite trend (Fig 2C). The percentage of genes related to posttranslational modification, protein turnover and chaperones (PMTC) decrease, whereas that of genes related to cytoskeleton increases over the course of the gonadotrophic cycle (Fig 2C).

Although MT genes account for most of the EMGs and LMGs, genes that belong to different functional sub-categories seem to be more prevalent in each gene set. While genes related to inorganic ion transport and metabolism (IIT&M) make up 25% of the early-mid MT genes, lipid transport and metabolism (LT&M) accounts for a similar amount of the late-mid MT genes (Fig 2D).

It is worth mentioning that in each of the four gene sets, ~40% of the genes belong to diverse or unknown functional classes, defined as having either insufficient information or no significant matches to other organisms.

### EGs activated by AAs and JH but repressed by 20E

Next, we investigated the regulatory signaling network responsible for the temporal dynamics of gene expression in the fat body during the PBM period of the mosquito gonadotrophic cycle. Previous studies have identified involvement of AAs, insulin and 20E in regulation of vitellogenic events in the mosquito fat body [15, 16, 24]. We used a combination of RNA interference (RNAi) and *in-vitro* organ culture techniques to elucidate the regulation of the temporal gene expression program in the fat body.

**Fig 3:**
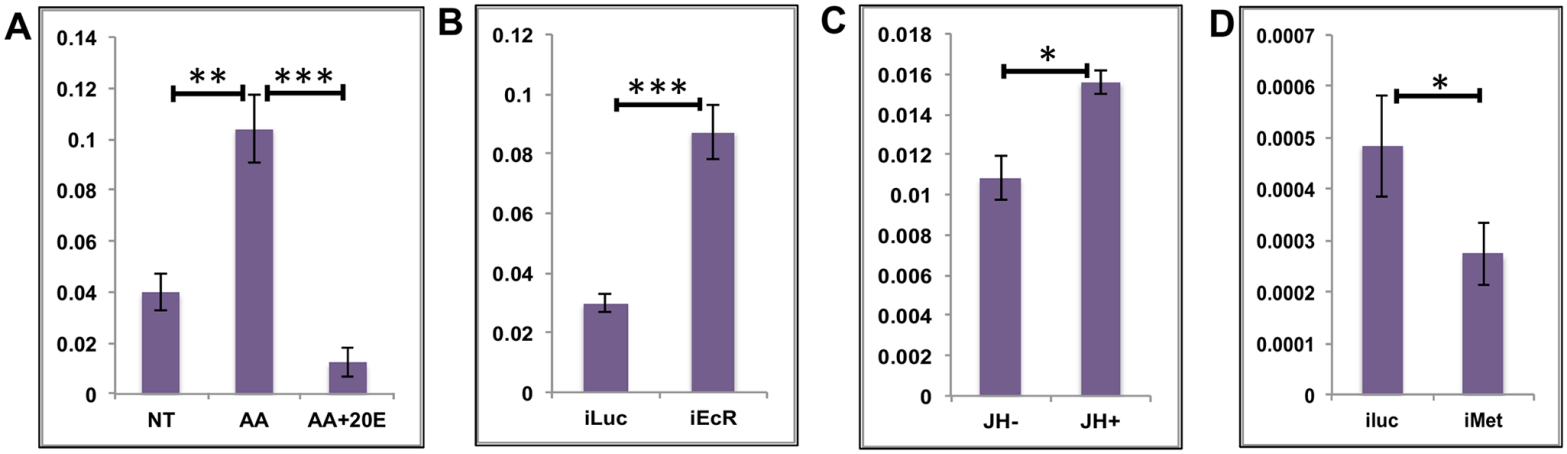
Effects of AAs, 20E and JH on representative EGs. **(A)** Relative expression of AAEL002269, Purine nucleoside phosphorylase detected by qRT-PCR, in tissues subjected to *in-vitro* fat body culture (IVFBC) in culture media without (NT) and with amino acids (AA) and with amino acid plus 20E (AA+20E) **(B)** Relative expression of the same gene detected by qRT-PCR, in fat body tissues collected from female mosquitoes post EcR knock-down (iEcR). **(C)** Relative expression of the same gene in tissues subjected to IVFBC in culture media without (JH-) and with (JH+) juvenile hormone. **(D)** Relative expression detected by qRT-PCR, in fat body tissues collected from female mosquitoes after knock-down of the JH receptor Met (imet). Injecting double stranded RNA for the Luciferase gene (iluc) served as the control in the RNAi experiments (**B** and **D**). All expressions calculated against housekeeping gene RPS7. Data representative of three biological replicates, with three technical replicates and are illustrated as average ± SD, * P < 0.05; ** P < 0.01; *** P < 0.001.

The average EG expression increased soon after a blood meal and reached its maximum between 6 h and 12 h PBM, just before the 20E titer started to peak (Fig 1C). The expression levels declined sharply with the increase in 20E titer, suggesting an inverse correlation between the 20E titer and EG expression. This is unlike the early genes in the 20E regulatory cascade where a positive correlation can be observed. We monitored the responses of three EGs (AAEL002269 –purine nucleoside phosphorylase; AAEL002488 –dead box ATP-dependent RNA helicase; and AAEL004345 –cysteinyl t-RNA synthetase) to treatments in *in-vitro* fat body culture (IVFBC) using qRT-PCR. The genes were selected on the basis of their high level of expression and similarity to the average profile (Fig 1C). Two of the three genes were IS&P genes, one from the RPM and one from the TRB sub-categories while the third was a nucleotide metabolism and transport gene. Each of these genes showed a differential expression of >2-fold. Tissues (fat bodies) collected at 72 h PE, when there is only a basal level of 20E, were placed in complete culture media and treated with either AAs alone or with AAs plus increasing concentrations of 20E (5x 10^−8^ M for 4 h and 10^−6^ M for 4 h); non-treated (NT) fat bodies in culture media served as the control. Total RNA was extracted, cDNA was made and qRT-PCR for the three EGs suggested that these genes were being upregulated by AAs and repressed by 20E (Fig 3A and S3A and S3B Fig).

To confirm the repression of these genes by 20E, we used the RNA-interference (RNAi) technique. Double-stranded RNA (dsRNA) was injected at 24 h PE to knock down EcR (S4A Fig), mosquitoes were blood fed 72 h post injection, and tissue was collected 24 h PBM. If these genes are being repressed by 20E then knocking down its receptor should remove the effects of repression. qRT-PCR for these genes with cDNA made from tissues collected from the EcR knocked-down mosquitoes confirmed that these genes are indeed repressed by 20E (Fig 3B and S3C and S3D Fig). Injecting dsRNA for the Luciferase gene (iLuc) served as the control.

Although the elevation of JH titer during the late PBM period has been reported (S1 Fig; [25, 26]), the role of this hormone in transcriptional regulation during the PBM period in the female mosquito is not entirely clear. To understand whether JH could play any regulatory role in EG expression during the late PBM period, we examined the effect of JH on the same genes using IVFBC and found that it had a moderate activating effect (Fig 3C and S3E and S3F Fig). The tissue used to check the effects of JH was collected at 24h PBM when the titer of JH was at the basal level, and incubated in a complete culture medium supplemented with either JH (10 μg/ml JH III) or the solvent (acetone) for 8 h.

To confirm this JH action, we knocked down the JH receptor, Met, by injecting a dsRNA (S4B Fig). Injections were done at 72 h PE, after the completion of the first preparatory phase; mosquitoes were blood fed 72 h post-injections and tissue was collected 72 h PBM. qRT-PCR for the same genes corroborated the activation by JH through its receptor Met, when the results demonstrated a decline in expression of the genes as a result of the Met knockdown (Fig 3D and S3G and S3H Fig). Next, we checked whether these genes were being activated by insulin, which has been reported to have a regulatory effect along with AAs on certain genes PBM [16, 17, 19, 27]. Exogenous insulin along with 20E has been shown to enhance AA-dependent activation of Vg expression in the isolated fat body [16]. The results suggested that insulin was not involved in the activation of these genes during the early PBM period.

Overall, our results have shown that the representatives of the EGs tested are activated by AAs at the early stage of the PBM period, are repressed by 20E/EcR in the mid stage and are activated again moderately by JH/Met at the end of the PBM phase. Involvement of insulin in the regulation of EGs tested could not be detected.

### EMGs are activated by 20E and repressed by HR3

**Fig 4:**
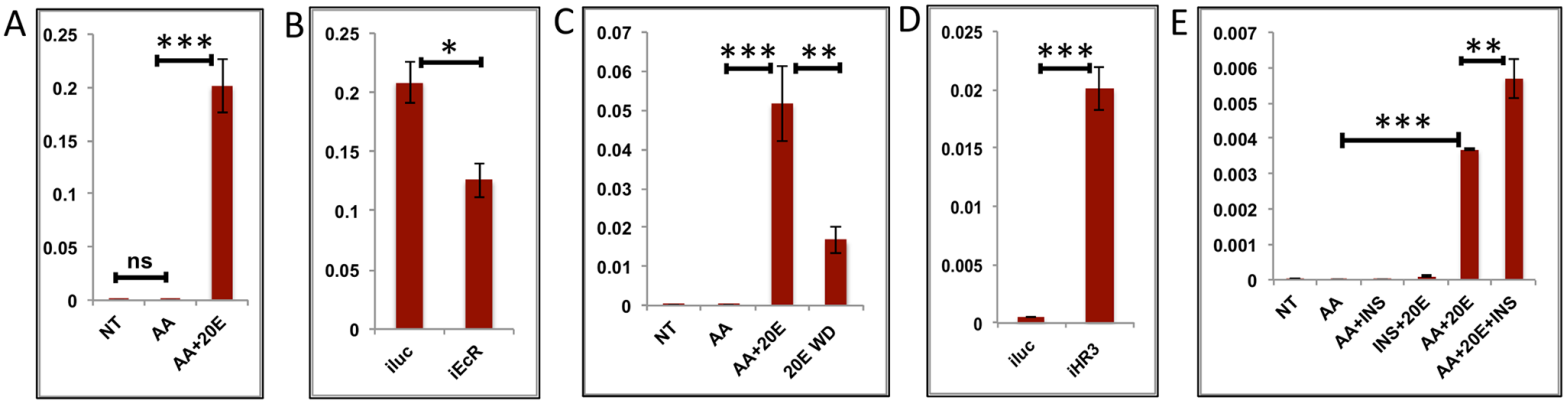
Effects of AAs, 20E, insulin and HR3 on YPP genes. **(A)** Relative expression of gene—AAEL006563, Carboxypeptidase, detected by qRT-PCR, in tissues subjected to *in-vitro* fat body culture (IVFBC) in culture media without (NT) and with amino acids (AA) and with amino acid plus 20E (AA+20E) **(B)** Relative expression of the same gene detected by qRT-PCR, in fat body tissues collected from female mosquitoes post EcR knock-down (iEcR).**(C)** Relative expression in tissues subjected IVFBC in culture media without (NT) and with amino acids (AA), with amino acids plus 20E (AA+20E) and after the withdrawal of 20E (20E WD). **(D)** Relative expression detected by qRT-PCR, in fat body tissues collected from female mosquitoes post HR3 knock-down (iHR3). **(E)** Relative expression of the same gene in tissues subjected to IVFBC in culture media without (NT) and with amino acids (AA), with amino acids and Insulin (AA+INS), Insulin and 20E (INS+20E), amino acids plus 20E (AA+20E), and amino acids plus 20E and Insulin (AA+20E+INS). Injecting double stranded RNA for the Luciferase gene (iluc) served as the control in the RNAi experiments (**B** and **D**). All expression calculated against housekeeping gene RPS7. Data representative of three biological replicates, with three technical replicates and are illustrated as average ± SD, * P < 0.05; ** P < 0.01; *** P < 0.001.

**Fig 5:**
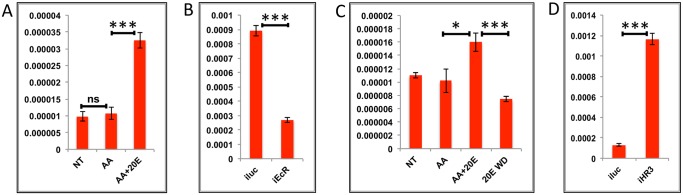
Effects of AAs, 20E and HR3 on EMGs. **(A)** Relative expression of gene—AAEL001433, fgf receptor activating protein detected by qRT-PCR, in tissues subjected to *in- vitro* fat body culture (IVFBC) in culture media without (NT) and with amino acids (AA) and with amino acid plus 20E (AA+20E) **(B)** Relative expression of the same gene detected by qRT-PCR, in fat body tissues collected from female mosquitoes post EcR knock-down (iEcR).**(C)** Relative expression in tissues subjected to IVFBC in culture media without (NT) and with amino acids (AA), with amino acids plus 20E (AA+20E) and after the withdrawal of 20E (20E WD). **(D)** Relative expression detected by qRT-PCR, in fat body tissues collected from female mosquitoes post HR3 knock-down (iHR3). Injecting double stranded RNA for the Luciferase gene (iluc) served as the control in the RNAi experiments (**B** and **D**). All expression calculated against housekeeping gene RPS7. Data representative of three biological replicates, with three technical replicates and are illustrated as average ± SD, * P < 0.05; ** P < 0.01; *** P < 0.001.

Transcript levels of the early-mid genes (EMGs) started increasing by 12 h, reached their maximum between 18 h and 24 h, and then declined drastically to basal levels by 36 h, staying low thereafter (Fig 1C). These genes show a positive correlation with the 20E titer during the PBM period. To examine the regulation of EMGs, we selected six genes, three well-known YPP genes (AAEL010434—Vitellogenin, AAEL007585—Cathepsin b and AAEL006563—Vitellogenic carboxypeptidase) and three others (AAEL014671- protease S51 alpha-aspartyl dipeptidase; AAEL001433 –FGF receptor activating protein; AAEL004398 –G-protein-coupled receptor). The latter genes were chosen on the basis of high expression and their similarity to the average EMG profile. Like the YPP genes, these three genes belong to the CP&S functional group and were upregulated by >8-fold. The expressions of genes were tested in the IVFBC with either AAs alone or AAs plus increasing concentrations of 20E for 8 h. The results showed that there is either minimal or no effect of AAs alone on these genes; however, all of these genes were activated by 20E in the presence of AAs (Figs 4A and 5A and S5A and S5B and S6A and S6B). To confirm the activation by 20E, these genes were tested using qRT-PCR with tissues (fat bodies) from EcR knocked-down mosquitoes (similar to those used for testing the EGs). The results corroborated that these genes are activated by 20E, as there was a decrease in transcript level for each of these genes in fat bodies from EcR-silenced mosquitoes (Figs 4B and 5B and S5C and S5D and S6C and S6D). The declines in these gene transcript levels correlate with the 20E titer drop in female mosquitoes, by about 30 h PBM. To test whether 20E was required for maintaining a high level of expression of EMGs, we modeled the 20E titer decrease in the IVFBC: the fat bodies from female mosquitoes 24h PBM were pre-incubated in the culture medium supplemented with AAs and changing concentration of 20E for 8 h (4 h each with two different concentrations, as described previously) and then incubated in a medium depleted of 20E for 3 h (three washes with complete culture medium every hour). The qRT-PCR results with cDNA made from these tissues showed a decline in the transcript levels of all six genes, further confirming the direct correlation between the 20E titer and the expression these genes (Figs 4C and 5C and S5E and S5F and S6E and S6F).

Expression levels of EMGs decline to their lowest levels by 36 h PBM when the orphan nuclear receptor HR3 has been reported to regulate transcriptional reprogramming of the fat body [28]. Therefore, we examined a possible effect of HR3 on these genes by RNAi interference. dsRNA was injected at 24 h PE to knock down HR3 (S4C Fig), the mosquitoes were blood fed 72 h post-injection and tissue was collected 36 h PBM. This RNAi experiment showed that the transcript levels of all tested genes were elevated, suggesting that they are indeed repressed by HR3 (Figs 4D and 5D and S5G and S5H and S6G and S6H). Therefore, as judged by testing six selected EMGs, the decrease in 20E titer and repression by HR3, constituted conditions responsible for the programmed decline in the transcriptional activity of EMGs.

It has been shown that insulin activates the YPP gene Vitellogenin in the presence of AAs and 20E in the *A*. *aegypti* fat body [16]. Our results confirmed a positive effect of insulin on expression of two other YPP genes, Vitellogenic carboxypeptidase (Fig 4E), and Cathepsin b (S5I Fig), along with that on the expression of Vitellogenin (S5J Fig). Surprisingly, we could not detect any activation by insulin of the other EMGs tested.

Since, the JH titer has been reported to rise again during the late PBM period, we wanted to check whether these genes are repressed by JH. The results suggested that JH has no repressive effect on these genes.

In summary, our findings indicate that representatives of the EMGs are activated by 20E and EcR, are downregulated by a declining 20E titer, and repressed by HR3. We also observed that insulin activated only a subset of EMGs, as tested here using the YPP genes.

### LMGs are repressed by 20E and JH but activated by HR3

**Fig 6:**
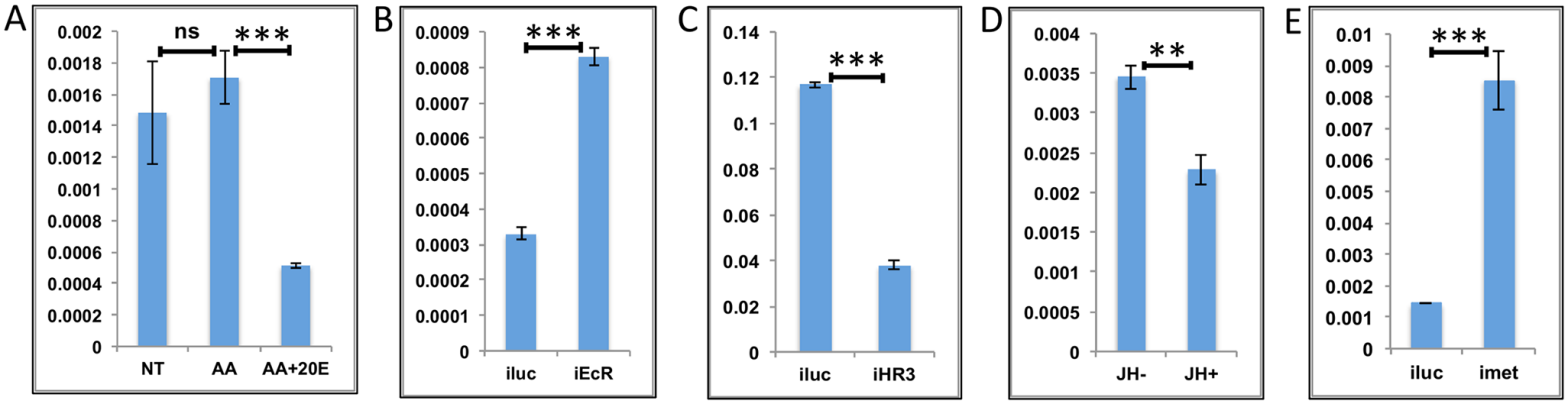
Effects of AAs, 20E, JH and HR3 on representative LMGs. **(A)** Relative expression of genes—AAEL003568, Threonine dehydratase detected by qRT-PCR, in tissues subjected to *in-vitro* fat body culture (IVFBC) in culture media without (NT) and with amino acids (AA) and with amino acid plus 20E (AA+20E) **(B)** Relative expression of the same gene detected by qRT-PCR, in fat body tissues collected from female mosquitoes post EcR knock-down (iEcR). **(C)** Relative expression detected by qRT-PCR, in fat body tissues collected from female mosquitoes post HR3 knock-down (iHR3). **(D)** Relative expression in tissues subjected to IVFBC in culture media without (JH-) and with (JH+) juvenile hormone. **(E)** Relative expression detected by qRT-PCR, in fat body tissues collected from female mosquitoes post Met knock-down (iMet). Injecting double stranded RNA for the Luciferase gene (iluc) served as the control in the RNAi experiments (**B**, **C** and **E**). All expression calculated against housekeeping gene RPS7. Data representative of three biological replicates, with three technical replicates and are illustrated as average ± SD, * P < 0.05; ** P < 0.01; *** P < 0.001.

The LMGs are a group in which the expressions of most of the genes are at low levels until 24 h PBM, after which they increase sharply and reach the maximal level between 36 h and 48 h PBM, declining thereafter (Fig 1C). Therefore, these genes appear to have a high level of expression only within a window when the titers of both 20E and JH are at low levels. We selected three genes on the basis of their high level of expression and similarity to the average LMG profile. All three genes (AAEL003568 –threonine dehydratase, AAEL010075—oxidoreductase and AAEL002638 –cytochrome 450) are metabolism-related genes and >10-fold upregulated at 36 h PBM when compared with their expression levels at 72 h PE. The effect of AAs *in-vitro* on these genes was not entirely consistent, because two of three genes tested were not affected (Fig 6A and S7A Fig), whereas the third (S7B Fig) showed activation by AAs. 20E repressed these genes *in-vitro*, which is consistent with their low level of expression up to 24 h PBM (Fig 6A and S7A and S7B Fig). EcR RNAi silencing (performed similarly to that described in the previous sections) confirmed the repression of these genes by EcR (Fig 6B and S7C and S7D Fig). Transcripts of the LMGs are elevated at the time when HR3 has been reported to be active in the mosquito fat body [28]. We hypothesized that HR3 is the factor responsible for upregulation of this gene set. When we conducted the HR3 RNAi silencing (as in previous section), it was indeed found that this nuclear receptor is responsible for the activation of the LMG representatives in the fat body (Fig 6C and S7E and S7F Fig). The LMGs showed a low level of expression between 48 h and 72 h PBM; therefore, we checked the effects of JH on the LMG representatives by IVFBC and found that these were repressed by JH (Fig 6D and S7G and S7H Fig). Met RNAi silencing revealed that these genes are repressed by JH through its receptor Met (Fig 6E and S7I and S7J Fig).

Overall, the results suggest that the LMG expression peak between 36 h and 48 h can likely be defined by repressive actions of 20E during the early PBM part and JH during the late PBM period. LMGs are activated by the reprogramming factor HR3 when the titers of both hormones are at relatively low levels.

### Activation of LGs by JH during the late PBM period

**Fig 7:**
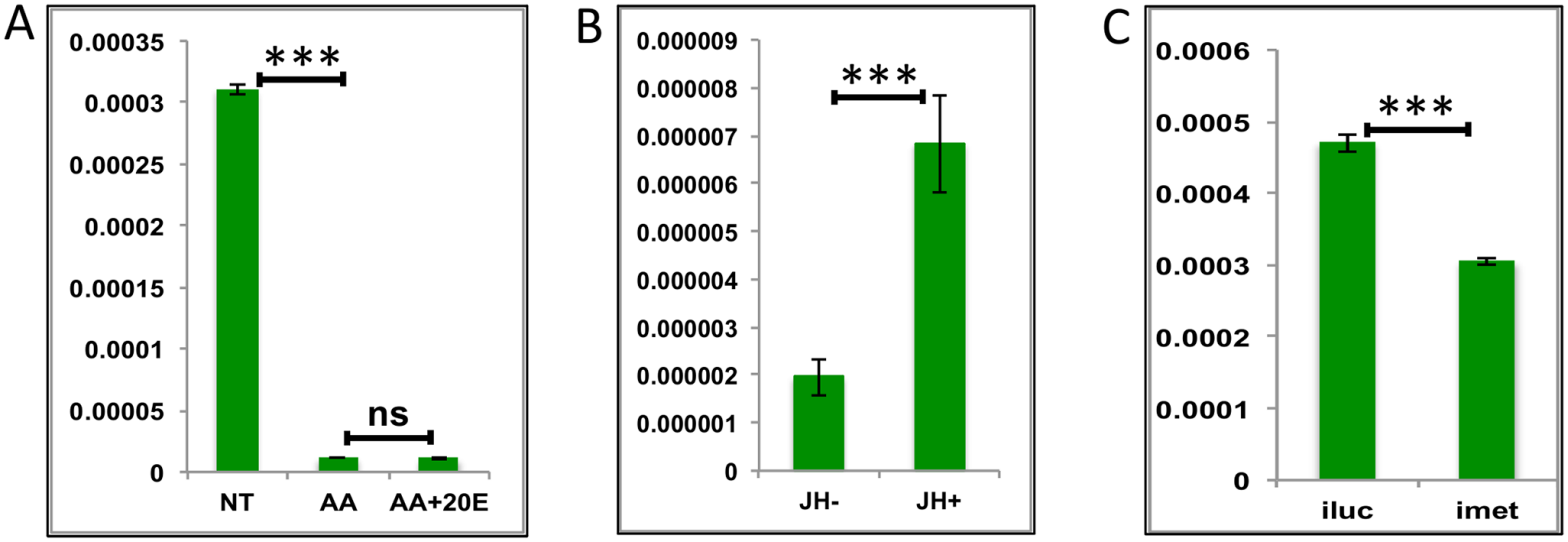
Effects of AAs, 20E and JH on representative LGs. **(A)** Relative expression of gene—AALE004328 –Origin recognition complex, detected by qRT-PCR, in tissues subjected to *in-vitro* fat body culture (IVFBC) in culture media without (NT) and with amino acids (AA) and with amino acid plus 20E (AA+20E) **(B)** Relative expression of the same gene in tissues subjected to IVFBC in culture media without (JH-) and with (JH+) juvenile hormone. **(C)** Relative expression detected by qRT-PCR, in fat body tissues collected from female mosquitoes post Met knock-down (iMet). Injecting double stranded RNA for the Luciferase gene (iluc) served as the control in the RNAi experiment. All expressions calculated against housekeeping gene RPS7. Data representative of three biological replicates, with three technical replicates and are illustrated as average ± SD, * P < 0.05; ** P < 0.01; *** P < 0.001.

The expression level of LGs starts to decline after a blood meal and remains low through the early-mid and late-mid phases PBM, before rising after 36 h and reaching maximal levels between 48 h and 72 h (Fig 1C). This pattern of LG expression has a positive correlation with the reported titer of JH during the PBM period [25, 26]. Our hypothesis was that 20E and JH determined temporal coordination of LG expression in which they are repressed by 20E during most of the PBM period and then activated by the rising titer of JH. In order to test this hypothesis, we selected three representative LGs (AAEL015143 –Glycine-rich ribosome binding protein; AAEL003352 –Ribosomal protein l7ae_E2; and AALE004328 –Origin recognition complex). We used the criteria described in the previous sections of high levels of expression and similarity to the average profile. All three genes were related to information storage and processing and were >20-fold downregulated when their minimum expressions were compared with their expressions at 72 h PE. Interestingly, IVFBC results showed all genes tested were downregulated by AAs and none by 20E (Fig 7A and S8A and S8B Fig). RNAi silencing of EcR corroborated that 20E does not have any repressive effect on the genes tested. In contrast, all of these genes were indeed activated by JH *in-vitro* (Fig 7B and S8C and S8D Fig). *In-vivo* RNAi confirmed their activation by JH through its receptor Met (Fig 7C and S8E and S8F Fig).

Thus, our experiments have shown that JH and Met play roles of major regulators for LG activation during the late PBM period. The AA pathway appears to be a repressor of these genes. Surprisingly, we found no role for 20E in the regulation of the genes tested from this group.

### Sets of genes are cyclically up- or down-regulated by JH through Met

**Fig 8:**
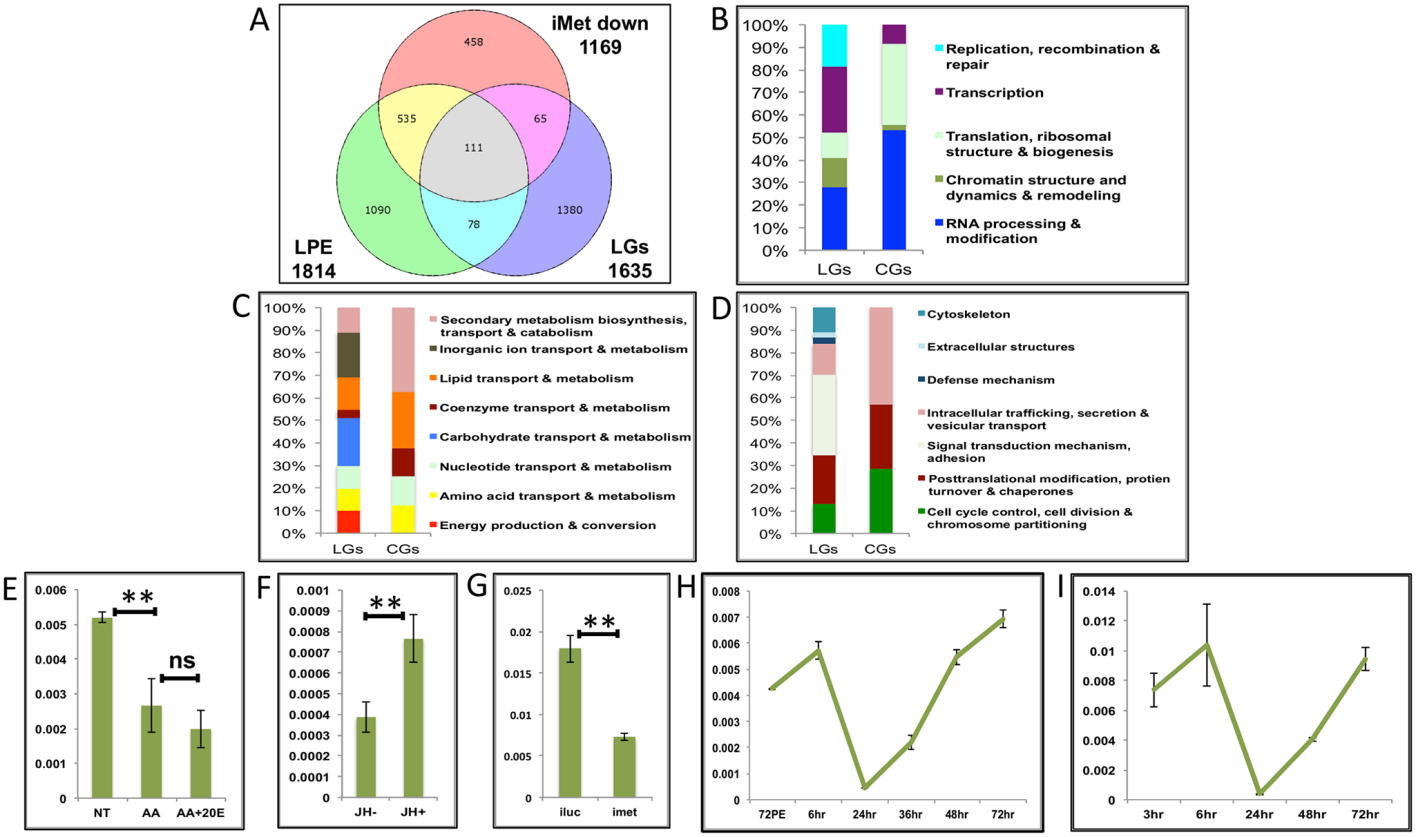
Genes cyclically activated by JH through Met—Functional group enrichment and the effects of AAs, 20E and JH. **(A)** Venn diagram showing genes that are up regulated in late post eclosion (LPE) and late post blood meal (LGs) periods and that are down regulated in Met knocked-down (imet down) fat body tissues. **(B-D)** Comparison of functional categories viz. **(B)** Information storage and Processing, **(C)** Metabolism and **(D)** Cellular Processes and Signalling, that constitute the late genes (LGs) and cyclical genes (CGs) using the inNOG database. **(E-F)** Relative expression of gene—AAEL001171, tRNA-dihydrouridine synthase detected by qRT-PCR, in tissues subjected to *in-vitro* fat body culture (IVFBC) in culture media, **(E)** without (NT) and with amino acids (AA) and with amino acid plus 20E (AA+20E); **(F)** without (JH-) and with (JH+) juvenile hormone. **(G)** Relative expression of the same gene detected by qRT-PCR, in fat body tissues collected from female mosquitoes post Met knock-down (iMet); injecting double stranded RNA for the Luciferase gene (iluc) served as the control. **(H)** Expression profile of the gene after the first blood meal. **(I)** Expression profile of the gene after the completion of the first reproductive cycle (egg laying) and post second blood meal. All expression calculated against housekeeping gene RPS7. Data representative of three biological replicates, with three technical replicates and are illustrated as average ± SD, * P < 0.05; ** P < 0.01; *** P < 0.001.

A characteristic feature of female mosquito reproduction is that it is cyclical, with each egg developmental cycle tightly linked to a separate blood feeding. Hence, we investigated whether the same genes are expressed during the late PE (LPE) and the late PBM periods, when the organism is preparing itself for a blood meal. We compared the LGs (all differentially expressed late genes based on the microarray data) with those expressed during the late PE period and are regulated by Met (LPE and iMet genes) [14]. We found that 111 late genes (S1 Table) appeared in both LPE (i.e., those are upregulated by >1.75-fold during the LPE period) and iMet downregulated (i.e., knockdown of Met during the PE period results in downregulation of >1.75-fold) gene sets (Fig 8A) and will be referred to as cyclical genes (CGs). Comparisons of functional groups constituting the LGs and CGs shows that—enrichment of the functional sub-categories in the two gene sets are markedly different (Fig 8B–8D and see S3 Dataset). We selected one gene each from the IS&P (AAEL001171- tRNA-dihydrouridine synthase), CP&S (AAEL002675 –arginase) and MT (AAEL001623—proteasome subunit) functional groups from within the 111 CGs and checked the effects of AAs, 20E and JH using IVBFC. IVBFC with AAs and changing concentrations of 20E demonstrated that these genes are repressed by AAs *in-vitro* (Fig 8E and S9A and S9B Fig), just like the PBM-specific LGs, whereas 20E may (S9A Fig) or may not (Fig 8E and S9B Fig) have a repressive effect. The activation by JH was evident from the IVFBCs with JH (Fig 8F and S9C and S9D Fig) and was confirmed by the *in-vivo* RNAi knock-down of the JH receptor Met (Fig 8G and S9E and S9F Fig). It is worth mentioning that Met dsRNA was injected at 72 h PE, after the completion of PE preparatory phase, and its effect was examined at 72 h PBM. We also found that one gene (AAEL003352) of the three, tested as PBM LGs, appeared in the list of LPE genes and was regulated by Met during the PE period.

Next, we checked the expression profiles of the representatives of CGs along with that of AAEL003352, post first (Fig 8H and S9G and S9H Fig) and second blood meal (Fig 8I and S9I and S9J Fig), after the mosquitoes completed the first reproductive cycle and had laid eggs. The expression profiles (Fig 8H and 8I and S9G–S9J Fig) demonstrated that these genes are indeed cyclical and are activated late during the second egg maturation cycle.

**Fig 9:**
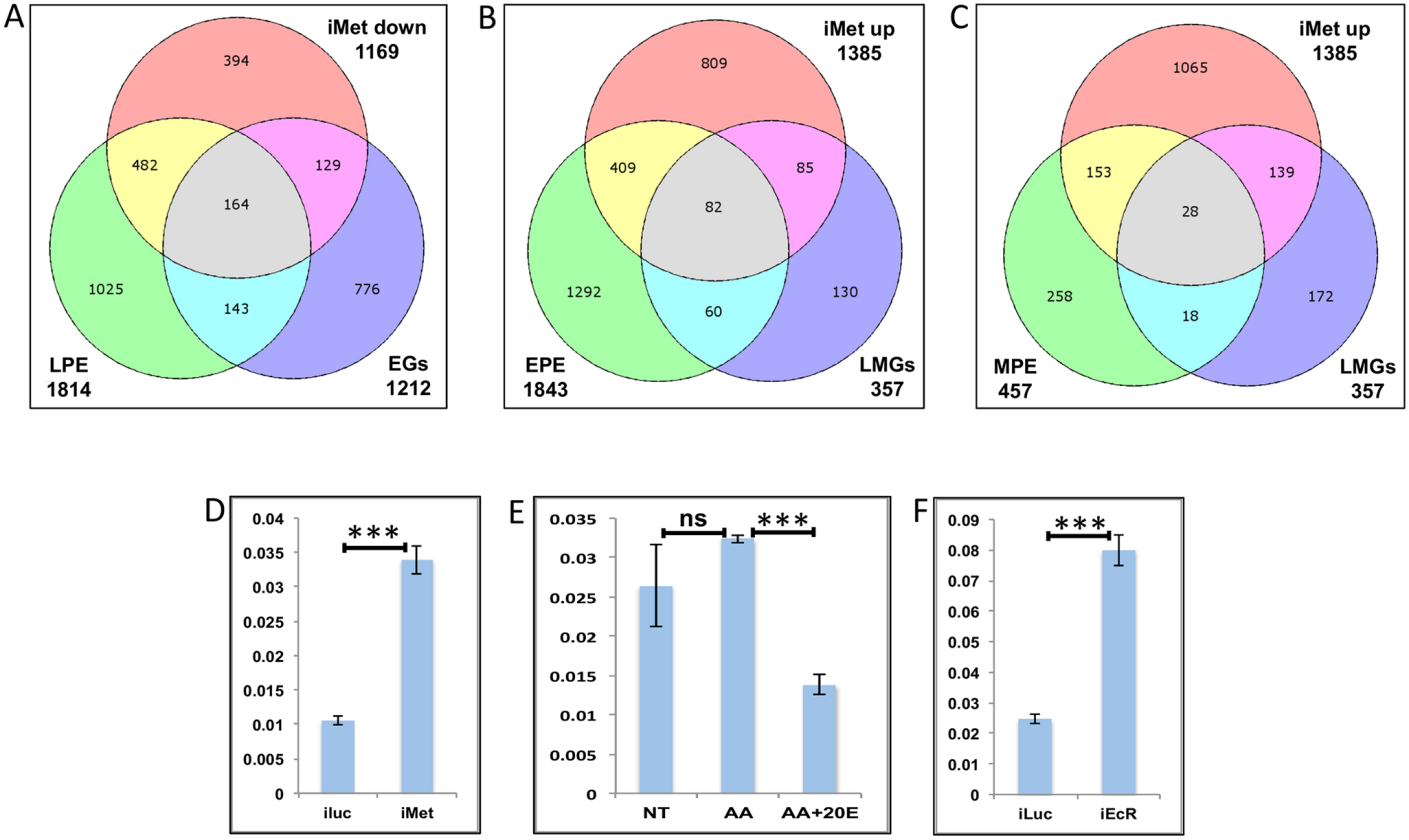
Genes cyclically regulated by JH through Met—the effects of AAs, 20E, EcR and Met on genes cyclically repressed by Met. **(A)** Venn diagram showing genes that are up regulated in late post eclosion (LPE) and early post blood meal (EGs) periods and that are down regulated in Met knocked-down (imet down) fat body tissues. **(B)** Venn diagram showing genes that are up regulated in early post eclosion (EPE) and late-mid post blood meal (LMGs) periods and that are up regulated in Met knocked-down (imet up) fat body tissues. **(C)** Venn diagram showing genes that are up regulated in early post eclosion (EPE) and late-mid post blood meal (LMGs) periods and that are up regulated in Met knocked-down (imet up) fat body tissues. **(D)** Relative expression of the gene, AAEL002781, Galactokinase detected by qRT-PCR, in fat body tissues collected from female mosquitoes post Met knock-down (iMet); injecting double stranded RNA for the Luciferase gene (iluc) served as the control. **(E)** Relative expression of the same gene detected by qRT-PCR, in tissues subjected to *in-vitro* fat body culture (IVFBC) in culture media without (NT) and with amino acids (AA) and with amino acid plus 20E (AA+20E). **(F)** Relative expression of the same gene detected by qRT-PCR, in fat body tissues collected from female mosquitoes post EcR knock-down (iEcR).

Our data suggests that EGs too can be up-regulated by JH to a lesser extent, probably at a later stage, therefore we compared the EGs with LPE and found that there are 164 genes that are common between the LPE, EGs and iMet-down sets (Fig 9A and S1 Table).

We also looked at the overlap between LMGs, representatives of which were found to be repressed by JH through Met, and the EPE and MPE sets, which according to Zou et al. [14], have a significant overlap with the iMet up (Met-repressed) sets and found 82 (Fig 9B and S1 Table) and 28 (Fig 9C and S1 Table) genes in common, respectively. We tested three of these genes (AAEL002781–Galactokinase, AAEL003347-CRAL/TRIO domain containing protein and AAEL000705 –Steroid dehydrogenase), common within the EPE, LMGs and iMet up sets, to check if these were repressed by JH through Met post blood meal. The qRT-PCR results with Met depleted samples (knock-down done at 72 h PE after the completion of the PE period) showed that these genes like in PE (as reported by Zou et al., 2013) are repressed by Met during the late-mid PBM period (Figs 9D and S10A–S10B). Since these are LMGs we tested the effects of AAs, 20E and EcR knockdown on these genes. The results showed that like in other LMGs, AAs may (S10C Fig) or may not (Figs 9E and S10D) have an effect on these genes, also these genes are repressed by 20E through EcR (Figs 9E and 9F and S10C–S10F).

In summary, the results suggest that there are sets of genes that are cyclically activated and repressed through the JH/Met pathway during both PE and PBM periods.

## Discussion

In this study, we have shown that certain key elements of the regulatory network mediating temporal gene expression in the fat body of a female mosquito, during blood-meal-activated reproduction, have dominant effects during specific periods. Unlike previous studies of the *A*. *aegypti* fat body transcriptome [29, 30] that have identified the differentially expressed genes at a single time point (24 h PBM), in this study, we have not only looked at the changes in the transcripts over a 72-h period (nine different time points) PBM, but have also demonstrated that there are key factors responsible for the differential expression of the genes. Our results have revealed the complexity of gene regulation in the fat body of female *A*. *aegypti* during this period, when the organism is undergoing massive physiological changes within a short time span. The inNOG database search reflects changes in the expression of genes that belong to the different functional groups during the four different stages (early, early-mid, late-mid and late) within the PBM period, with MT genes being highly active during the early- and late- mid PBM periods, between 18 h and 48 h, when blood is digested, yolk proteins are made and the fat body reprogramming for the next egg developmental cycle begins.

**Fig 10:**
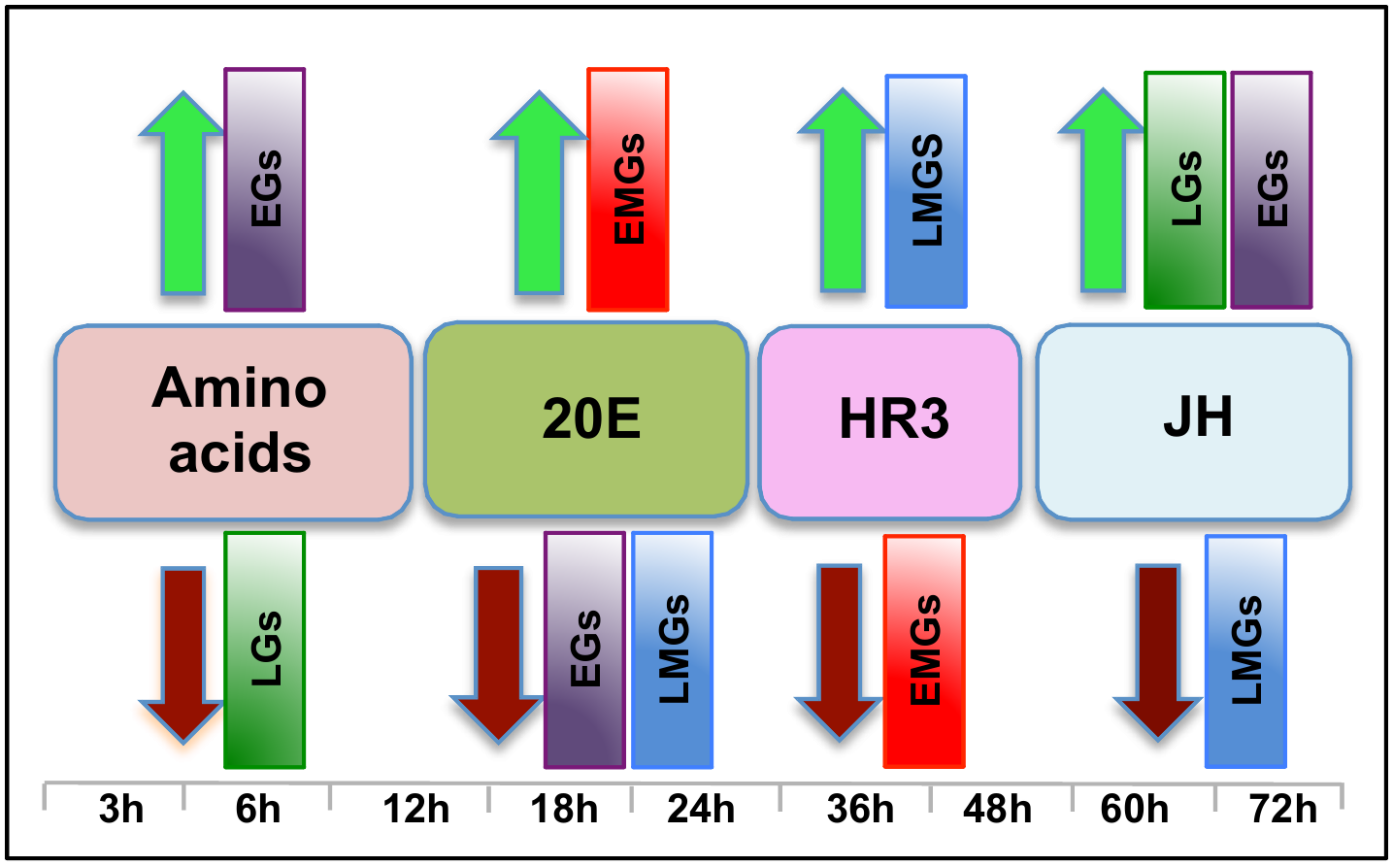
The main regulators mediating transcription in four distinct waves of gene expression during the gonadotrophic cycle in the fat body of female *Aedes aegypti*. Amino acids are the main regulators of gene expression during the early hours post blood meal (PBM); found to up- and down-regulate representatives of early (EGs) and late genes (LGs) respectively. Around 12 h PBM, the effect of 20E becomes apparent. 20E activates early mid genes (EMGs) and represses EGs as well as late mid genes (LMGs) between 18 h—24 h. HR3 seems to be the main regulator between 36 h and 48 h, the expressions of EMGs decline under the influence of HR3 whereas that of the LMGs increase. JH regulates the terminal part of this cycle by up-regulating late genes (LGs) and down-regulating the LMGs. A moderate activating effect of JH can be detected in case of the EGs during this period.

It has been well established that 20E is the major stimulus that upregulates YPP gene expression in mosquitoes, which, along with protein synthesis, positively correlates with 20E titers [20, 31]. In this study, we were able to demonstrate 20E-mediated activation of not only the YPP genes but also representatives of a super-group (EMGs) within which the YPPs fall (Fig 10). We have also shown that 20E represses representatives from groups of genes that are activated before the rise of the 20E titer (EGs), and after the decline of the 20E titer (LMGs) We also checked the response of representative genes from both of these groups to a lower concentration of 20E and found that there is either no effect or varying degrees of repressive effects (S11A–S11F Fig). To the best of our knowledge, this is the first study to report large-scale transcriptional repression by 20E and EcR. While the molecular mechanism of gene activation by 20E hierarchy is well established, little is known about repressive 20E action.

Although 20E is known to be the main regulator of vitellogenesis, by itself it is not sufficient to activate this process. Experiments have demonstrated that AAs are critical for the activation of YPP genes by 20E [15, 31], and we have shown here that AAs are not only essential for the activation of YPP genes by 20E, but can activate and repress gene sets without the assistance of 20E. Representatives of EGs were found to be activated, whereas those of LGs were found to be repressed by AAs (Fig 10).

Around 36 h PBM, when the titer of 20E has declined to basal levels and YPP synthesis has ceased, the fat body converts back to a nutrient storage and metabolism function until the next vitellogenic cycle is initiated [32]. It has been demonstrated previously that HR3 targets genes in the termination phase of the vitellogenic cycle and plays an essential role in programmed termination of the first cycle as well as in the entry into the second [28]. In this study, we demonstrated that the repressive effects of HR3 are not limited to vitellogenin or even the YPP genes. During the termination of vitellogenesis HR3 inhibits a good number of genes, mostly EMGs (Fig 10), which are activated by 20E between 18 h and 24 h PBM. Similarly, activation by HR3 is not restricted to the transcription factors in the 20E regulatory cascade, but affects a much larger set of genes (mostly LMGs) that are activated during the termination of vitellogenesis, between 36 h and 48 h (Fig 10). These effects of HR3 might either be direct or indirect through other transcription factors activated by HR3. For example, HR3 stimulates transcription of genes encoding EcRB and USPA isoforms [28]. In turn, they may participate in mediating HR3 action. This question requires further investigation. Thus, the critical role played by HR3 during the termination of the first vitellogenic cycle by switching on and off certain gene sets has been well established in this study.

JH is associated with changes in the fat body, during the pre-vitellogenic period, which allows the fat body to become responsive to signals that induce vitellogenesis [33–35]. The fat body becomes competent to respond to the steroid hormone 20E and to synthesize the massive amounts of yolk protein required for egg maturation [36]. In this study, we have identified the role of JH in transcriptional regulation during the late PBM (post vitellogenic) period and demonstrated that JH not only activates representatives of a group of genes (LGs), but also is responsible for repressing genes that are activated by HR3 during the termination of vitellogenesis (LMGs, Fig 10). Moreover, apart from activating genes in the late PBM period, it also regulates a set of genes (CGs) that are cyclical. The genes that are cyclically activated by JH follow the JH titer and probably play important roles in preparing the fat body for successive vitellogenic cycles. There are also genes that are cyclically repressed through Met during both PE and PBM periods. Thus, this work demonstrates that the role of JH in transcriptional regulation is not limited to the pre-vitellogenic stage; along with HR3, JH plays a key role in remodeling the fat body for the next egg developmental cycle, post vitellogenesis.

In contrast to observed effects of AAs, 20E, HR3 and JH, we were unable to detect any large-scale effects of insulin, other than activation of the YPP genes, within the fat body, during the PBM period. We were able to detect a synergistic activating effect of 20E and insulin in the presence of amino acids (previously reported by Roy et al. 2007) on genes tested other than Vitellogenin. Surprisingly, the other EMGs (of which YPPs is a subgroup) tested showed no significant activation by insulin. We also did not detect any activation of the EGs by insulin, either by itself or in the presence of amino acids and 20E, suggesting that activation by insulin is limited to the YPP genes during the early and early-mid PBM period.

Therefore, we can summarize, based on the microarray transcriptomic analysis that the PBM period can be divided broadly into four phases: early (0–12 h), early-mid (12-30h), late-mid (30–48h) and late (48-72h). Our study using *in-vitro* organ culture and RNAi depletion analyses also revealed the major regulators of gene expression during these four phases—AAs, 20E, HR3 and JH, respectively (Fig 10). Each of these factors is responsible for the activation and repression of different gene sets during the four distinct PBM phases, thereby successfully completing vitellogenesis, and the reprogramming of the fat body, for the next reproductive cycle (Fig 10). These results provide a clear insight into the complexity of gene regulation within this key mosquito tissue, thereby elucidating the coordination among the different key regulators in the orchestration of spatial and temporal gene expression patterns required during this critical phase of female mosquito reproduction. In-vitro fat body culture has its limitations as the dissected tissue loses communication with other tissues and therefore cannot receive the signals arising from those tissues. We have in most cases checked the effects of single factor at one time, and utilized RNAi mediated depletion of complementary factors for this analyses. The goal of this study was to identify how the major regulators affect the waves of expression during a specific time period. Although we have accomplished this primary goal, further study is required to identify other factors involved in the regulation of gene expression during these periods.

## Materials and Methods

### Mosquito rearing

Mosquitoes of the *A*. *aegypti* wild-type UGAL strain were raised at 27°C and 80% humidity, as described previously [16]. The larvae were raised in non-crowded conditions [37,38] (200 in 750 ml distilled water per 9” x 12” pan) and fed 0.125 ml– 0.900 ml vol. of standard diet (equal parts of rodent diet, Lactalbumin and active dry yeast) between Day 0 and Day 4. Four pans of pupae were combined into one adult cage. Adult mosquitoes were fed continuously on water and 10% (wt/vol) sucrose solution. All dissections were performed in Aedes physiological solution (APS) at room temperature [16]. Blood feeding of all adult mosquitoes other than the Met knocked-down ones, was done with white rats. Adult Met knocked-down mosquitoes were blood-fed using White Leghorn chickens. All procedures for using vertebrate animals were approved by the UCR animal care and use committee.

### Microarray assays

Single-color hybridizations of custom-made Agilent microarrays with 15,321 *A*. *aegypti* genes were conducted at the University of Chicago Core Instrument Facility following standard protocols [22]. Three independent biological replicates were performed for each treatment. The raw image data were processed using the Agilent Feature Extraction Software [39]. Subsequent analysis steps including background correction, normalization and statistical analysis of DEGs, were performed in the statistical programming environment R using Bioconductor packages [40], as described previously [14]. DEGs were filtered and hierarchical clustering was performed using the same criteria used previously [14]. Discrete clusters were obtained by the same tree cutting approach described before [14].

### eggNOG database search

The dataset for insects non-supervised orthologous groups (inNOG) and their proteins, from the eggNOG v3.0 (evolutionary genealogy of genes: Non-supervised Orthologous Groups) database was used for determination of the functional categories. 11318 *Aedes aegypti* genes are present in version 3.0 of the inNOG dataset that was downloaded. The EGs, EMGs, LMGs, LGs and CGs were mapped against the inNOG dataset with the help of the Microsoft (MS) Acess software. MS Excel was used to generate the stacked column bar graphs.

### *In-vitro* fat-body culture

#### For testing the effects of AAs, 20E and insulin

A total of 9–12 fat bodies were dissected from mosquitoes 72 h PE and incubated in a complete culture medium [16] supplemented with amino acids [16] and increasing concentrations of 20E (Sigma, St. Louis, MO; 5x 10^−8^ M for 4 h and 10^−6^ M for 4h) for 8 h (S12 Fig), to check the effects of amino acids and 20E. To check the effects of lower concentration of 20E, tissues were incubated with AAs and increasing concentrations of 20E (1x 10^−8^ M for 4 h and 5x 10^−8^ M for 4h) Similar tissues were used to check the effects of insulin, using 17μM bovine insulin solution (Sigma, St. Louis, MO). Experiments were repeated three times under similar conditions.

#### For testing the effects of JH

Tissues were collected from female mosquitoes at 24 h PBM and were incubated with JH (Sigma, St. Louis, MO; 10 μg/mL JH III) or solvent (acetone) added to the culture medium for 8 h. Experiments were done in triplicate under the same conditions.

### Total RNA extraction

#### For microarray transcriptome analysis

RNA samples were collected at nine time points, starting at 3 h PBM. RNA was extracted from fat bodies of 9–12 female mosquitoes using the TRIzol method (Invitrogen, Carlsbad, CA) according to the manufacturer’s protocol. It was concentrated using the RNeasy MinElute cleanup kit (Qiagen, Valencia, CA) for further processing.

#### For qRT-PCR post IVFBC

RNA was extracted from 9–12 fat bodies of female mosquitoes post IVFBC, using the TRIzol method (Invitrogen) according to the manufacturer’s protocol.

### cDNA preparation

cDNAs were synthesized from 2 μg total RNA using the SuperScript III Reverse Transcriptase kit (Invitrogen). RNA was treated with DNase I (Invitrogen) before cDNA synthesis. PCR was performed using the Platinum High Fidelity Supermix (Invitrogen).

### dsRNA preparation and microinjection

#### For iEcR and iHR3

To synthesize EcR and HR3 dsRNA, we followed a method described previously [16]. In brief, dsRNA of a specific gene template was synthesized using the MEGAscript T7 kit (Ambion, Austin, TX) and the luciferase gene was used to generate control iLuc dsRNA. After dsRNA synthesis, samples were subjected to phenol/chloroform extraction and ethanol precipitation. dsRNA then was suspended in RNase-free water to a final concentration of 5 μg/μl. At 24 h PE, female mosquitoes were injected with 300 nl dsRNA into the thorax. The Picospritzer II (General Valve Corporation, Fairfield, NJ) was used to introduce corresponding dsRNAs into the thorax of CO_2_-anesthetized female mosquitoes.

The knockdown efficiencies for EcR ranged between 53.3% and 62.7% whereas that of HR3 ranged between 55.6% and 64.1%.

#### For iMet

To synthesize Met dsRNA, we followed the same method as for iEcR and iHR3, except that female mosquitoes were injected with 300 nl dsRNA into the thorax at 72 h PE, after the completion of the first preparatory cycle. The knockdown efficiencies of Met ranged between 40.3% and 53.2%.

Sequences of all primers used for dsRNA preparation are shown in S2 Table.

### qRT-PCR analysis

qRT-PCR was performed using the iCycler iQ system (Bio-Rad, Hercules, CA and an IQ SYBR Green Supermix (Bio-Rad). Quantitative measurements were performed in triplicate and relative expression (RE) was measured as RE = 2^-ΔΔCt^ and normalized to the internal control of S7 ribosomal protein mRNA for each sample. Real-time data were collected from the software iCycler v3.0. Raw data were exported to Microsoft Excel and analyzed. P-values were calculated with the help of unpaired t test using the online version of GraphPad.

Sequences of all primers used for qRT-PCR analyses are shown in S2 Table.

## Supporting Information

S1 Fig Differentially regulated genes over the course of the PBM period. Number of transcripts significantly upregulated and downregulated (left y-axis) in the microarray experiment [fold change ≥1.75 (0.8 in log2 scale) and a false-discovery rate (*P* value) of ≤0.01] in a chronological time order (h PBM; x-axis). 20E and JH titers (dashed lines), labeled on the right y-axis, are from Hagedorn et al. (S1 Reference) and Shapiro et al. [25], respectively.
(TIF)

S2 Fig Validation of the microarray data using qRT-PCR. **(A-C)** Comparison of microarray (blue) and qRT-PCR (red) expression data for representatives of the Early Genes (EGs) set that were used for further analysis. **(D-F)** Comparison of microarray (blue) and qRT-PCR (red) expression data for the three Yolk Protein Precursor genes, that were used as representatives of the Early-Mid Genes (EMGs) set for further analysis. **(G-I)** Comparison of microarray (blue) and qRT-PCR (red) expression data for representatives of the Late-Mid Genes (LMGs) set that were used for further analysis. **(J-L)** Comparison of microarray (blue) and qRT-PCR (red) expression data for representatives of the Late Genes (LGs) set that were used for further analysis. The X-axis depicts the log2-fold change when the expression is compared to that at 72h PE and the Y-axis represents the time-points for tissue collection.
(TIFF)

S3 Fig Effects of AAs, 20E and JH on representative EGs. **(A-B)** Relative expression of genes—AAEL002488, Dead box atp dependent RNA helicase and AAEL004345, Cysteinyl t-RNA synthetase, detected by qRT-PCR, in tissues subjected to *in-vitro* fat body culture (IVFBC) in culture media without (NT) and with amino acids (AA) and with amino acid plus 20E (AA+20E) **(C-D)** Relative expression of the same genes detected by qRT-PCR, in fat body tissues collected from female mosquitoes post EcR knock-down (iEcR). **(E-F)** Relative expression in tissues subjected to IVFBC in culture media without (JH-) and with (JH+) juvenile hormone. **(G-H)** Relative expression detected by qRT-PCR, in fat body tissues collected from female mosquitoes post Met knock-down (iMet). Injecting double stranded RNA for the Luciferase gene (iluc) served as the control in the RNAi experiments (**C-D** and **G-H**). All expressions calculated against housekeeping gene RPS7. Data representative of three biological replicates, with three technical replicates and are illustrated as average ± SD, * P < 0.05; ** P < 0.01; *** P < 0.001.
(TIFF)

S4 Fig Validation of knock-downs using qRT-PCR. Relative expression of **(A)** AAEL009600—EcR, **(B)** AAEL001746—Met and **(C)** AAEL009588—HR3 genes detected by qRT-PCR, in fat body tissues collected from female mosquitoes post **(A)** EcR, **(B)** Met and **(C)** HR3 knock-downs, respectively. Injecting double stranded RNA for the Luciferase gene (iluc) served as the control. All expression calculated against housekeeping gene RPS7. Data representative of three biological replicates, with three technical replicates and are illustrated as average ± SD, * P < 0.05; ** P < 0.01; *** P < 0.001.
(TIFF)

S5 Fig Effects of AAs, 20E, insulin and HR3 on YPP genes. (**A-B**) Relative expression of genes—AAEL007585, Cathepsin b and AAEL010434, Vitellogenin, detected by qRT-PCR, in tissues subjected to *in-vitro* fat body culture (IVFBC) in culture media without (NT) and with amino acids (AA) and with amino acid plus 20E (AA+20E). (**C-D**) Relative expression of the same genes detected by qRT-PCR, in fat body tissues collected from female mosquitoes post EcR knock-down (iEcR). (**E-F**) Relative expression in tissues subjected to IVFBC in culture media without (NT) and with amino acids (AA), with amino acids plus 20E (AA+20E) and after the withdrawal of 20E (20E WD). (**G-H**) Relative expression detected by qRT-PCR, in fat body tissues collected from female mosquitoes post HR3 knock-down (iHR3). (**I-J**) Relative expression of the same genes in tissues subjected to IVFBC in culture media without (NT) and with amino acids (AA), with amino acids plus Insulin (AA+INS), Insulin and 20E (INS+20E), amino acids plus 20E (AA+20E), and amino acids plus 20E and Insulin (AA+20E+INS). Injecting double stranded RNA for the Luciferase gene (iluc) served as the control in the RNAi experiments (**C-D** and **G-H**). All expressions calculated against housekeeping gene RPS7. Data representative of three biological replicates, with three technical replicates and are illustrated as average ± SD, * P < 0.05; ** P < 0.01; *** P < 0.001.
(TIF)

S6 Fig Effects of AAs, 20E and HR3 on EMGs. **(A-B**) Relative expression of genes—AAEL004398, g protein-coupled receptor and AAEL014671, protease S51 alpha-aspartyl dipeptidase detected by qRT-PCR, in tissues subjected to *in-vitro* fat body culture (IVFBC) in culture media without (NT) and with amino acids (AA) and with amino acid plus 20E (AA+20E) (**C-D**) Relative expression of the same genes detected by qRT-PCR, in fat body tissues collected from female mosquitoes post EcR knock-down (iEcR). (**E-F**) Relative expression in tissues subjected to IVFBC in culture media without (NT) and with amino acids (AA), with amino acids plus 20E (AA+20E) and after the withdrawal of 20E (20E WD). (**G-H**) Relative expression detected by qRT-PCR, in fat body tissues collected from female mosquitoes post HR3 knock-down (iHR3). Injecting double stranded RNA for the Luciferase gene (iluc) served as the control in the RNAi experiments (**C-D** and **G-H**). All expressions calculated against housekeeping gene RPS7. Data representative of three biological replicates, with three technical replicates and are illustrated as average ± SD, * P < 0.05; ** P < 0.01; *** P < 0.001.
(TIF)

S7 Fig Effects of AAs, 20E, JH and HR3 on representative LMGs. **(A-B)** Relative expression of genes—AAEL010075, oxidoreductase and AAEL002638, Cytochrome 450 detected by qRT-PCR, in tissues subjected to *in-vitro* fat body culture (IVFBC) in culture media without (NT) and with amino acids (AA) and with amino acid plus 20E (AA+20E) **(C-D)** Relative expression of the same genes detected by qRT-PCR, in fat body tissues collected from female mosquitoes post EcR knock-down (iEcR). **(E-F)** Relative expression detected by qRT-PCR, in fat body tissues collected from female mosquitoes post HR3 knock-down (iHR3). **(G-H)** Relative expression in tissues subjected to IVFBC in culture media without (JH-) and with (JH+) juvenile hormone. **(I-J)** Relative expression detected by qRT-PCR, in fat body tissues collected from female mosquitoes post Met knock-down (iMet). Injecting double stranded RNA for the Luciferase gene (iluc) served as the control in the RNAi experiments (**C-D, E-F and I-J**). All expression calculated against housekeeping gene RPS7. Data representative of three biological replicates, with three technical replicates and are illustrated as average ± SD, * P < 0.05; ** P < 0.01; *** P < 0.001.
(TIF)

S8 Fig Effects of AAs, 20E and JH on representative LGs. **(A-B)** Relative expression of genes—AAEL015143 –Glycine rich ribosome binding protein and AALE003352 –Ribosomal Protein l7ae_E2, detected by qRT-PCR, in tissues subjected to *in-vitro* fat body culture (IVFBC) in culture media without (NT) and with amino acids (AA) and with amino acid plus 20E (AA+20E) **(C-D)** Relative expression of the same genes in tissues subjected to IVFBC in culture media without (JH-) and with (JH+) juvenile hormone. **(E-F)** Relative expression detected by qRT-PCR, in fat body tissues collected from female mosquitoes post Met knock-down (iMet). Injecting double stranded RNA for the Luciferase gene (iluc) served as the control in the RNAi experiments. All expression calculated against housekeeping gene RPS7. Data representative of three biological replicates, with three technical replicates and are illustrated as average ± SEM, * P < 0.05; ** P < 0.01; *** P < 0.001.
(TIF)

S9 Fig Effects of AAs, 20E and JH on representative of genes cyclically activated by JH. **(A-B)** Relative expression of genes—AAEL002675, arginase and AAEL001623, proteasome subunit, detected by qRT-PCR, in tissues subjected to *in-vitro* fat body culture (IVFBC) in culture media without (NT) and with amino acids (AA) and with amino acid plus 20E (AA+20E) **(C-D)** Relative expression of the same genes in tissues subjected to IVFBC in culture media without (JH-) and with (JH+) juvenile hormone. **(E-F)** Relative expression of the same genes detected by qRT-PCR, in fat body tissues collected from female mosquitoes post Met knock-down (iMet). Injecting double stranded RNA for the Luciferase gene (iluc) served as the control in the RNAi experiments. **(G-H)** Expression profiles of the same genes post first blood meal. (**I-J**) Expression profiles of the genes after the completion of the first reproductive cycle (egg laying) and post second blood meal. All expression calculated against housekeeping gene RPS7. Data representative of three biological replicates, with three technical replicates and are illustrated as average ± SD, * P < 0.05; ** P < 0.01; *** P < 0.001.
(TIFF)

S10 Fig Effects of Met, AAs and 20E on representatives of genes cyclically repressed by JH. **(A-B)** Relative expression of the genes—AAEL003347, CRAL/TRIO domain containing protein and AAEL000705, Steroid dehydrogenase, detected by qRT-PCR, in fat body tissues collected from female mosquitoes post Met knock-down (iMet); injecting double stranded RNA for the Luciferase gene (iluc) served as the control. **(C-D)** Relative expression of the same genes detected by qRT-PCR, in tissues subjected to *in-vitro* fat body culture (IVFBC) in culture media without (NT) and with amino acids (AA) and with amino acid plus 20E (AA+20E). **(E-F)** Relative expression of the same genes detected by qRT-PCR, in fat body tissues collected from female mosquitoes post EcR knock-down (iEcR) injecting double stranded RNA for the Luciferase gene (iluc) served as the control.
(TIFF)

S11 Fig Effects of IVBFC with low concentration of 20E on representatives of EGs and LMGs. **(A-C)** Relative expression of the early genes—AAEL002269, Purine nucleoside phosphorylase (A), AAEL002488, Dead box atp dependent RNA helicase (B) and AAEL004345, Cysteinyl t-RNA synthetase (C) detected by qRT-PCR, in tissues subjected to *in-vitro* fat body culture (IVFBC) in culture media without (NT) and with amino acids (AA), with amino acid plus low concentration of 20E (AA+20E LO) and with amino acid plus high concentration of 20E (AA+20E HI). **(D-F)** Relative expression of the late-mid genes—AAEL002781, Galactokinase (D), AAEL003347, CRAL/TRIO domain containing protein (E) and AAEL000705, Steroid dehydrogenase (F) detected by qRT-PCR, in tissues subjected to *in-vitro* fat body culture (IVFBC) in culture media without (NT) and with amino acids (AA), with amino acid plus low concentration of 20E (AA+20E LO) and with amino acid plus high concentration of 20E (AA+20E HI).
(TIFF)

S12 Fig Validation of the method used for IVBFC with 20E. Genes respond significantly better when incubated with two different concentrations (5x 10^−8^ M and 10^−6^ M) of 20E, along with AAs (AA+20E) for 8 h (4 h + 4 h respectively), in IVBFC, than, when incubated for 8 h with the higher concentration of 20E plus AAs (AA+20E NC). The YPP genes–(A) Vitellogenin, (B) Cathepsin beta and (C) Carboxypeptidase were used for this test. Data representative of three biological replicates, with three technical replicates and are illustrated as average ± SD, * P < 0.05; ** P < 0.01; *** P < 0.001.
(TIF)

S1 Table List of transcripts cyclically up- and down- regulated by JH/Met. (XLSX)

S2 Table Primer sequences used for dsRNA preparation and qRT-PCR analyses. (XLSX)

S1 Dataset Normalized expressions of all transcripts showing differential expression at one or more of the nine time points, post blood meal, when compared to their expression at 72 h PE. (XLSX)

S2 Dataset Normalized expressions of Early- (EGs), Early-mid- (EMGs), Late-mid- (LMGs) and Late- (LGs) gene transcripts during the nine time points post blood meal. (XLSX)

S3 Dataset inNOG id, description and functional group assignments of all post blood meal, differentially expressed, *A*. *aegypti* genes that mapped to the inNOG dataset of the eggNOG v 3.0 database. (XLSX)

S1 Reference Supporting Information reference. (DOCX)
