# Supplementary figures and images for: Regulation of Gene Expression Patterns in Mosquito Reproduction
Source: PLoS Genet. 2015 Aug 14;11(8):e1005450. doi: 10.1371/journal.pgen.1005450 (PMC4537244; doi:10.1371/journal.pgen.1005450)

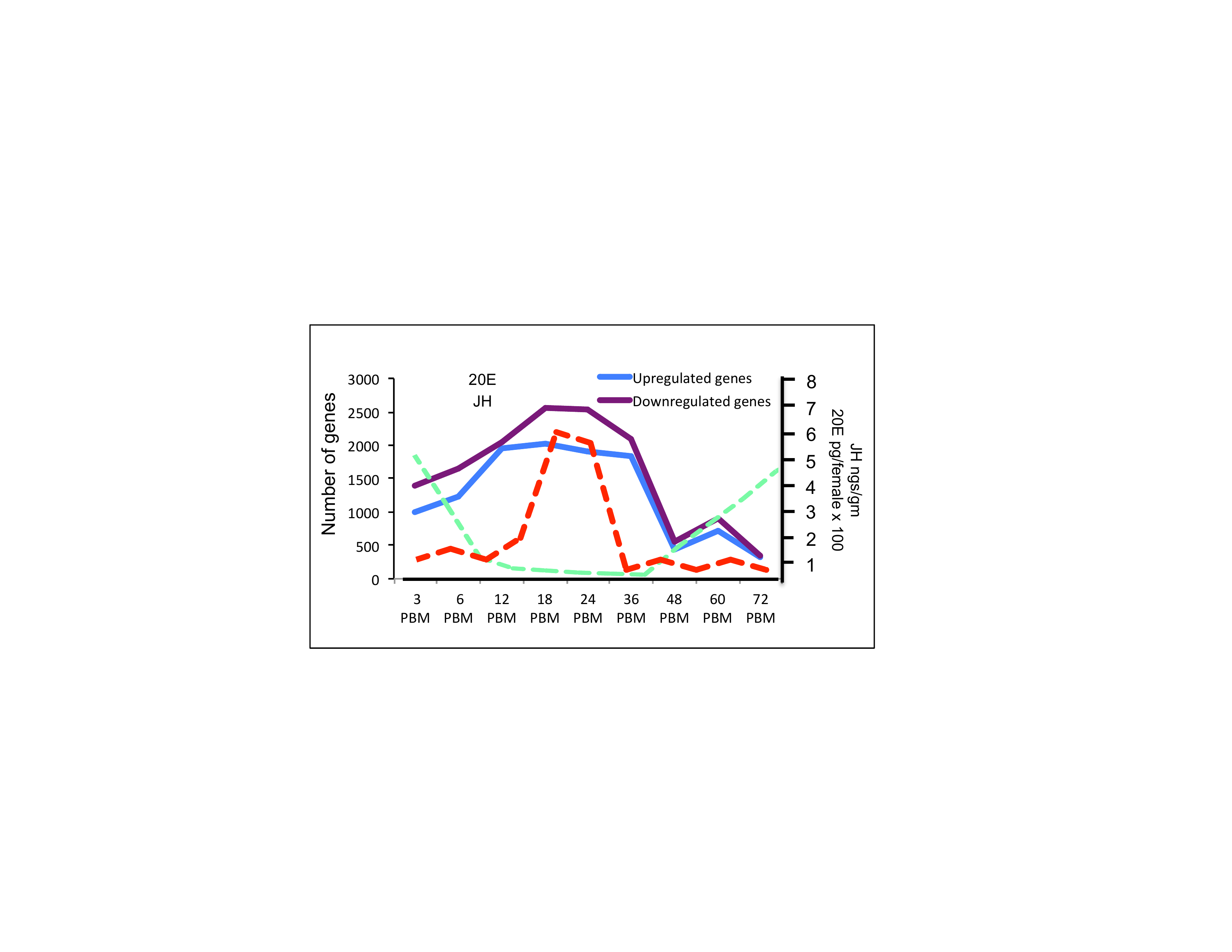

Supplement: S1 Fig — Number of transcripts significantly upregulated and downregulated (left y-axis) in the microarray experiment [fold change ≥1.75 (0.8 in log2 scale) and a false-discovery rate (P value) of ≤0.01] in a chronological time order (h PBM; x-axis). 20E and JH titers (dashed lines), labeled on the right y-axis, are from Hagedorn et al. (S1 Reference) and Shapiro et al. [25], respectively. (TIF) [file pgen.1005450.s001.tif]

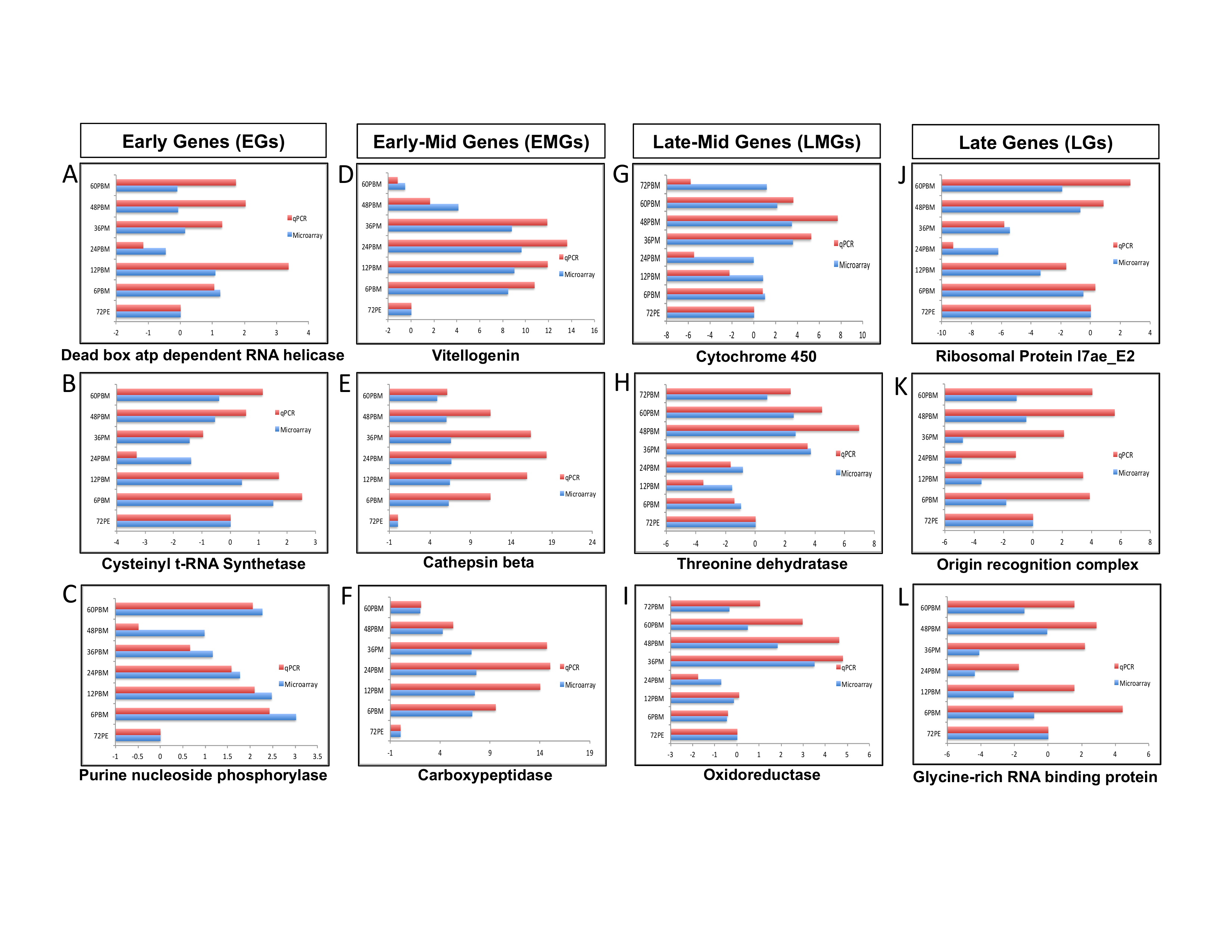

Supplement: S2 Fig — (A-C) Comparison of microarray (blue) and qRT-PCR (red) expression data for representatives of the Early Genes (EGs) set that were used for further analysis. (D-F) Comparison of microarray (blue) and qRT-PCR (red) expression data for the three Yolk Protein Precursor genes, that were used as representatives of the Early-Mid Genes (EMGs) set for further analysis. (G-I) Comparison of microarray (blue) and qRT-PCR (red) expression data for representatives of the Late-Mid Genes (LMGs) set that were used for further analysis. (J-L) Comparison of microarray (blue) and qRT-PCR (red) expression data for representatives of the Late Genes (LGs) set that were used for further analysis. The X-axis depicts the log2-fold change when the expression is compared to that at 72h PE and the Y-axis represents the time-points for tissue collection. (TIFF) [file pgen.1005450.s002.tiff]

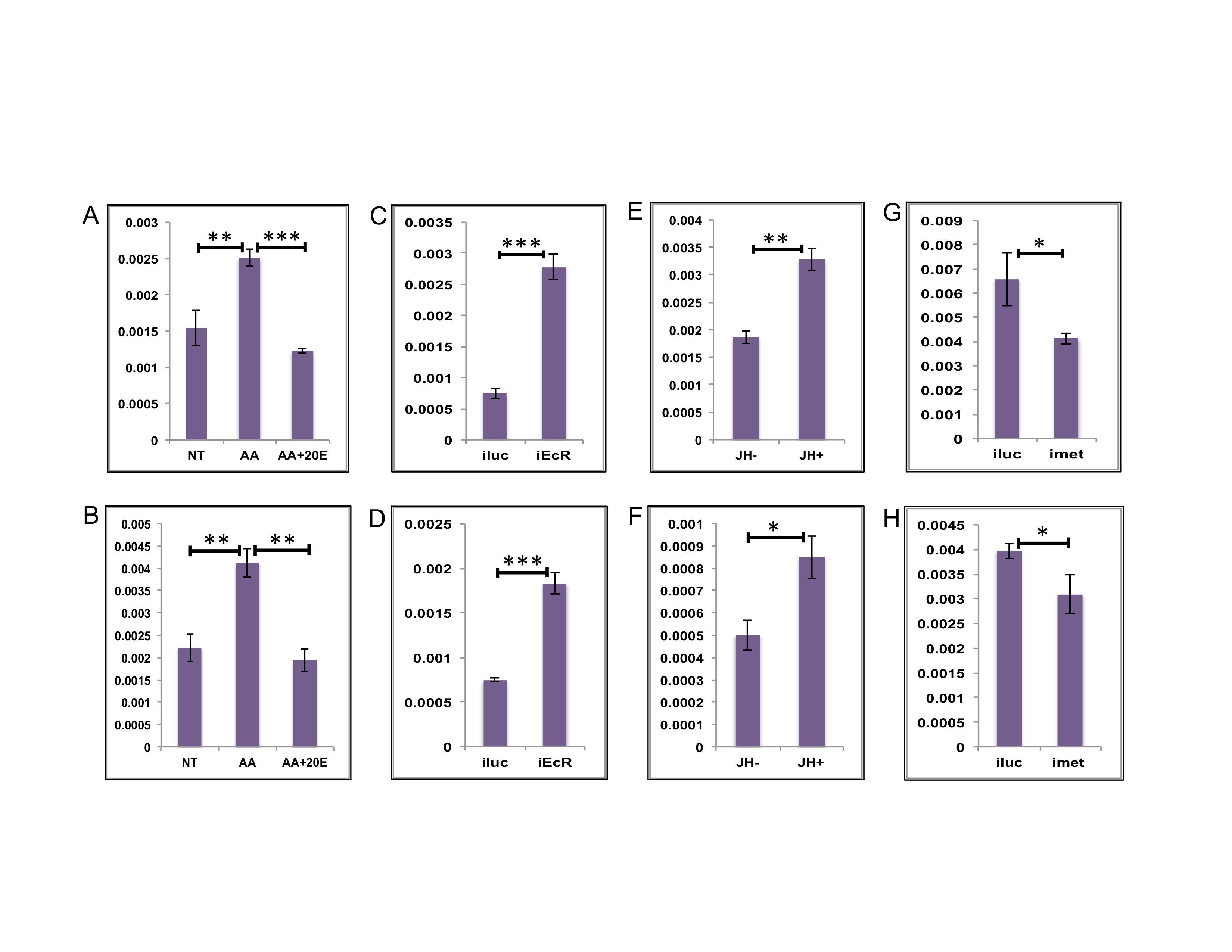

Supplement: S3 Fig — (A-B) Relative expression of genes—AAEL002488, Dead box atp dependent RNA helicase and AAEL004345, Cysteinyl t-RNA synthetase, detected by qRT-PCR, in tissues subjected to in-vitro fat body culture (IVFBC) in culture media without (NT) and with amino acids (AA) and with amino acid plus 20E (AA+20E) (C-D) Relative expression of the same genes detected by qRT-PCR, in fat body tissues collected from female mosquitoes post EcR knock-down (iEcR). (E-F) Relative expression in tissues subjected to IVFBC in culture media without (JH-) and with (JH+) juvenile hormone. (G-H) Relative expression detected by qRT-PCR, in fat body tissues collected from female mosquitoes post Met knock-down (iMet). Injecting double stranded RNA for the Luciferase gene (iluc) served as the control in the RNAi experiments (C-D and G-H). All expressions calculated against housekeeping gene RPS7. Data representative of three biological replicates, with three technical replicates and are illustrated as average ± SD, * P < 0.05; ** P < 0.01; *** P < 0.001. (TIFF) [file pgen.1005450.s003.tiff]

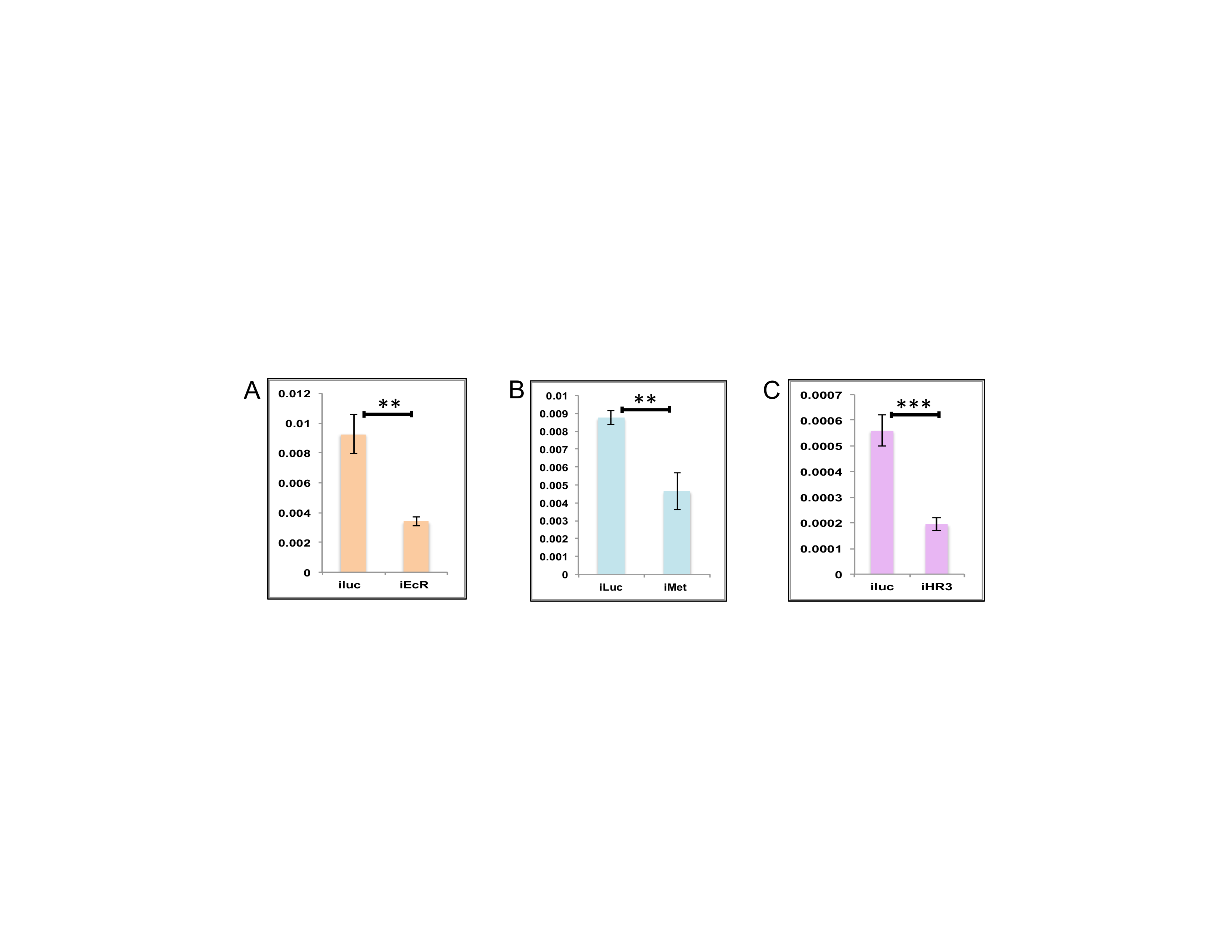

Supplement: S4 Fig — Relative expression of (A) AAEL009600—EcR, (B) AAEL001746—Met and (C) AAEL009588—HR3 genes detected by qRT-PCR, in fat body tissues collected from female mosquitoes post (A) EcR, (B) Met and (C) HR3 knock-downs, respectively. Injecting double stranded RNA for the Luciferase gene (iluc) served as the control. All expression calculated against housekeeping gene RPS7. Data representative of three biological replicates, with three technical replicates and are illustrated as average ± SD, * P < 0.05; ** P < 0.01; *** P < 0.001. (TIFF) [file pgen.1005450.s004.tiff]

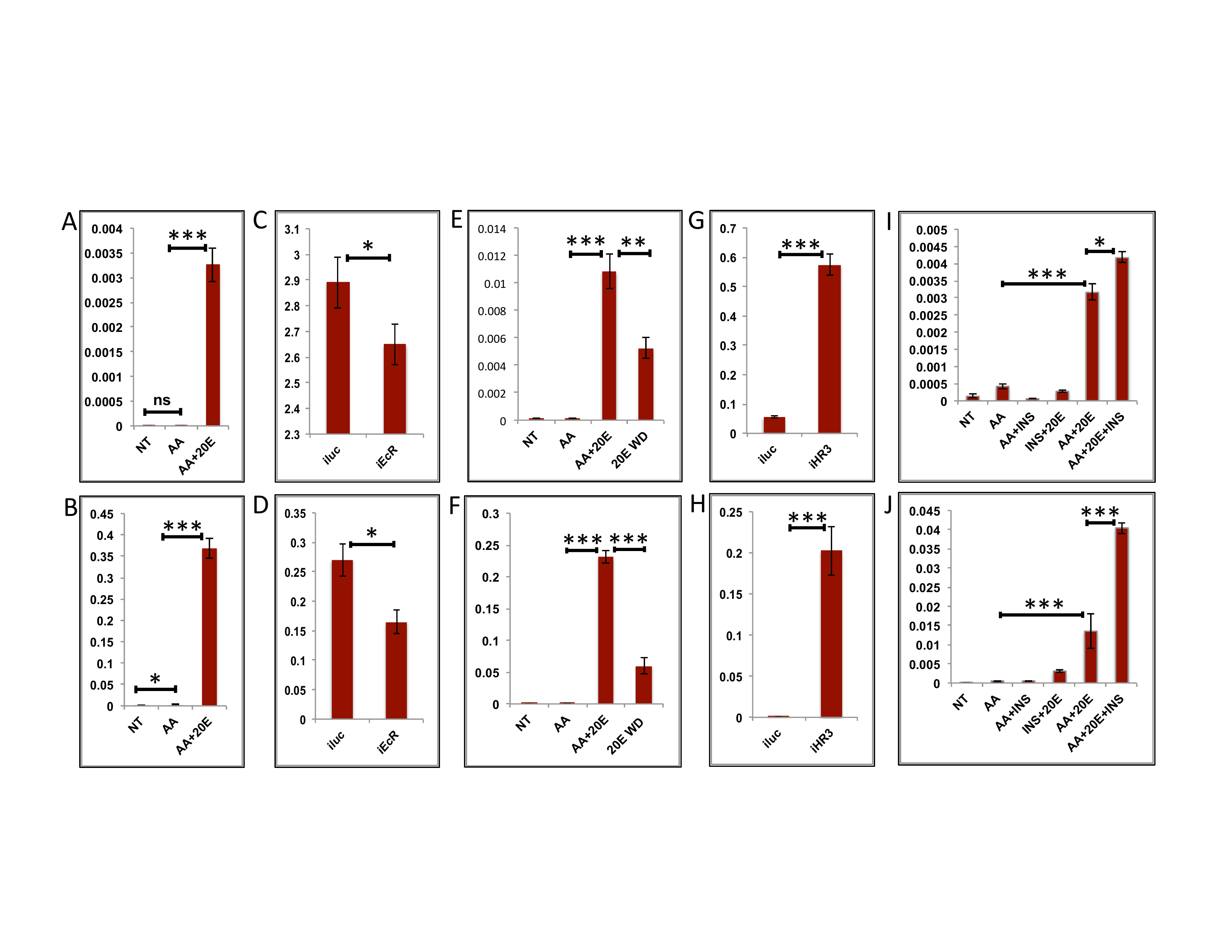

Supplement: S5 Fig — (A-B) Relative expression of genes—AAEL007585, Cathepsin b and AAEL010434, Vitellogenin, detected by qRT-PCR, in tissues subjected to in-vitro fat body culture (IVFBC) in culture media without (NT) and with amino acids (AA) and with amino acid plus 20E (AA+20E). (C-D) Relative expression of the same genes detected by qRT-PCR, in fat body tissues collected from female mosquitoes post EcR knock-down (iEcR). (E-F) Relative expression in tissues subjected to IVFBC in culture media without (NT) and with amino acids (AA), with amino acids plus 20E (AA+20E) and after the withdrawal of 20E (20E WD). (G-H) Relative expression detected by qRT-PCR, in fat body tissues collected from female mosquitoes post HR3 knock-down (iHR3). (I-J) Relative expression of the same genes in tissues subjected to IVFBC in culture media without (NT) and with amino acids (AA), with amino acids plus Insulin (AA+INS), Insulin and 20E (INS+20E), amino acids plus 20E (AA+20E), and amino acids plus 20E and Insulin (AA+20E+INS). Injecting double stranded RNA for the Luciferase gene (iluc) served as the control in the RNAi experiments (C-D and G-H). All expressions calculated against housekeeping gene RPS7. Data representative of three biological replicates, with three technical replicates and are illustrated as average ± SD, * P < 0.05; ** P < 0.01; *** P < 0.001. (TIF) [file pgen.1005450.s005.tif]

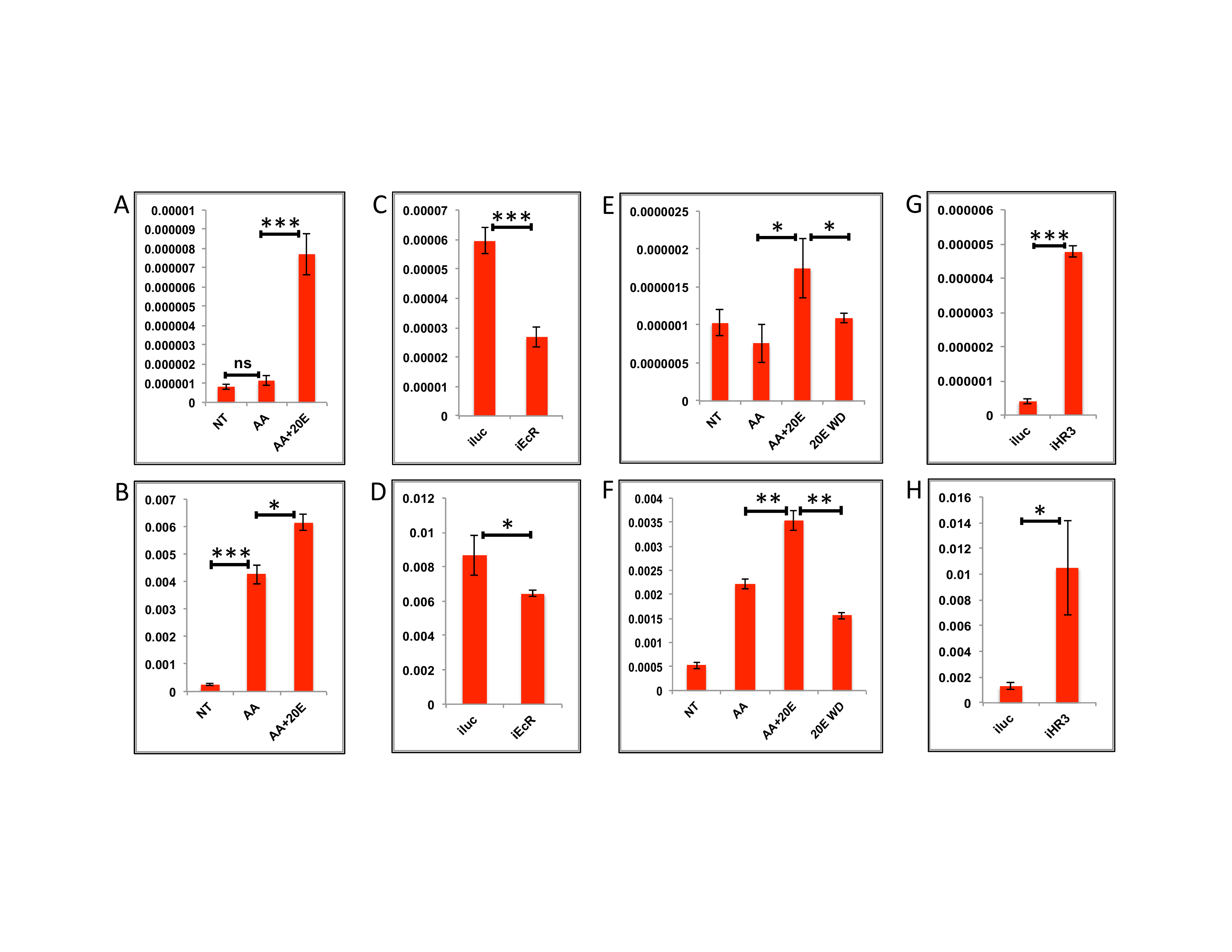

Supplement: S6 Fig — (A-B) Relative expression of genes—AAEL004398, g protein-coupled receptor and AAEL014671, protease S51 alpha-aspartyl dipeptidase detected by qRT-PCR, in tissues subjected to in-vitro fat body culture (IVFBC) in culture media without (NT) and with amino acids (AA) and with amino acid plus 20E (AA+20E) (C-D) Relative expression of the same genes detected by qRT-PCR, in fat body tissues collected from female mosquitoes post EcR knock-down (iEcR). (E-F) Relative expression in tissues subjected to IVFBC in culture media without (NT) and with amino acids (AA), with amino acids plus 20E (AA+20E) and after the withdrawal of 20E (20E WD). (G-H) Relative expression detected by qRT-PCR, in fat body tissues collected from female mosquitoes post HR3 knock-down (iHR3). Injecting double stranded RNA for the Luciferase gene (iluc) served as the control in the RNAi experiments (C-D and G-H). All expressions calculated against housekeeping gene RPS7. Data representative of three biological replicates, with three technical replicates and are illustrated as average ± SD, * P < 0.05; ** P < 0.01; *** P < 0.001. (TIF) [file pgen.1005450.s006.tif]

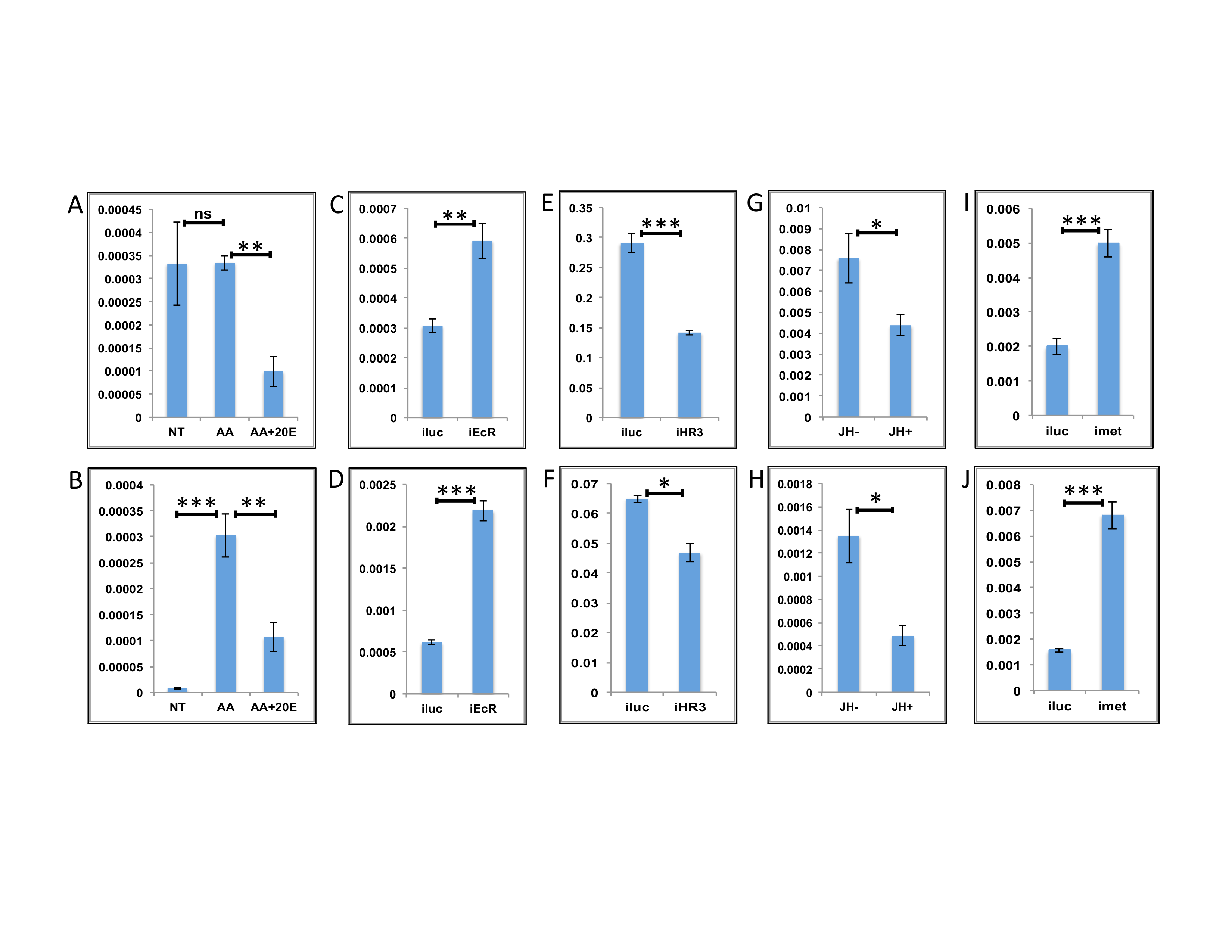

Supplement: S7 Fig — (A-B) Relative expression of genes—AAEL010075, oxidoreductase and AAEL002638, Cytochrome 450 detected by qRT-PCR, in tissues subjected to in-vitro fat body culture (IVFBC) in culture media without (NT) and with amino acids (AA) and with amino acid plus 20E (AA+20E) (C-D) Relative expression of the same genes detected by qRT-PCR, in fat body tissues collected from female mosquitoes post EcR knock-down (iEcR). (E-F) Relative expression detected by qRT-PCR, in fat body tissues collected from female mosquitoes post HR3 knock-down (iHR3). (G-H) Relative expression in tissues subjected to IVFBC in culture media without (JH-) and with (JH+) juvenile hormone. (I-J) Relative expression detected by qRT-PCR, in fat body tissues collected from female mosquitoes post Met knock-down (iMet). Injecting double stranded RNA for the Luciferase gene (iluc) served as the control in the RNAi experiments (C-D, E-F and I-J). All expression calculated against housekeeping gene RPS7. Data representative of three biological replicates, with three technical replicates and are illustrated as average ± SD, * P < 0.05; ** P < 0.01; *** P < 0.001. (TIF) [file pgen.1005450.s007.tif]

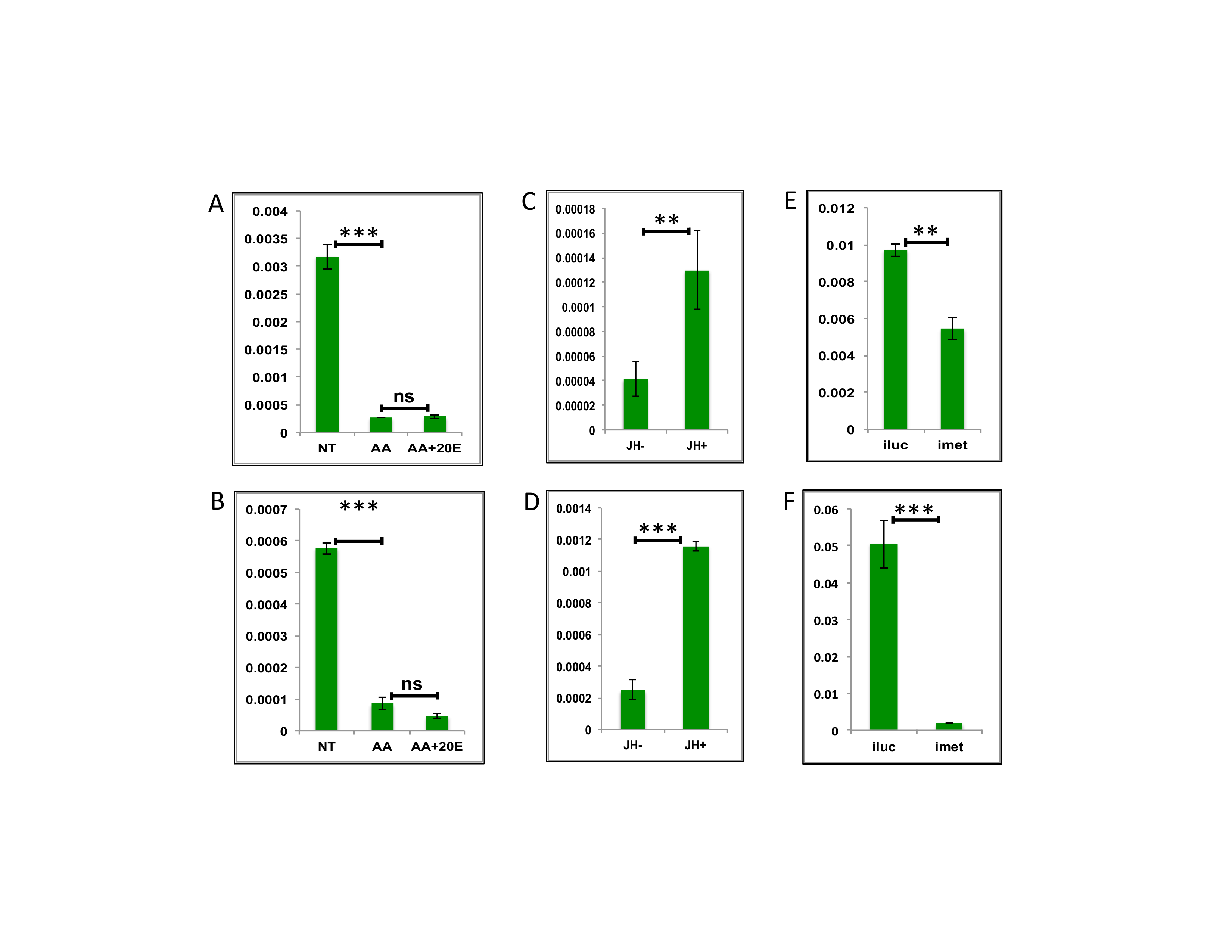

Supplement: S8 Fig — (A-B) Relative expression of genes—AAEL015143 –Glycine rich ribosome binding protein and AALE003352 –Ribosomal Protein l7ae_E2, detected by qRT-PCR, in tissues subjected to in-vitro fat body culture (IVFBC) in culture media without (NT) and with amino acids (AA) and with amino acid plus 20E (AA+20E) (C-D) Relative expression of the same genes in tissues subjected to IVFBC in culture media without (JH-) and with (JH+) juvenile hormone. (E-F) Relative expression detected by qRT-PCR, in fat body tissues collected from female mosquitoes post Met knock-down (iMet). Injecting double stranded RNA for the Luciferase gene (iluc) served as the control in the RNAi experiments. All expression calculated against housekeeping gene RPS7. Data representative of three biological replicates, with three technical replicates and are illustrated as average ± SEM, * P < 0.05; ** P < 0.01; *** P < 0.001. (TIF) [file pgen.1005450.s008.tif]

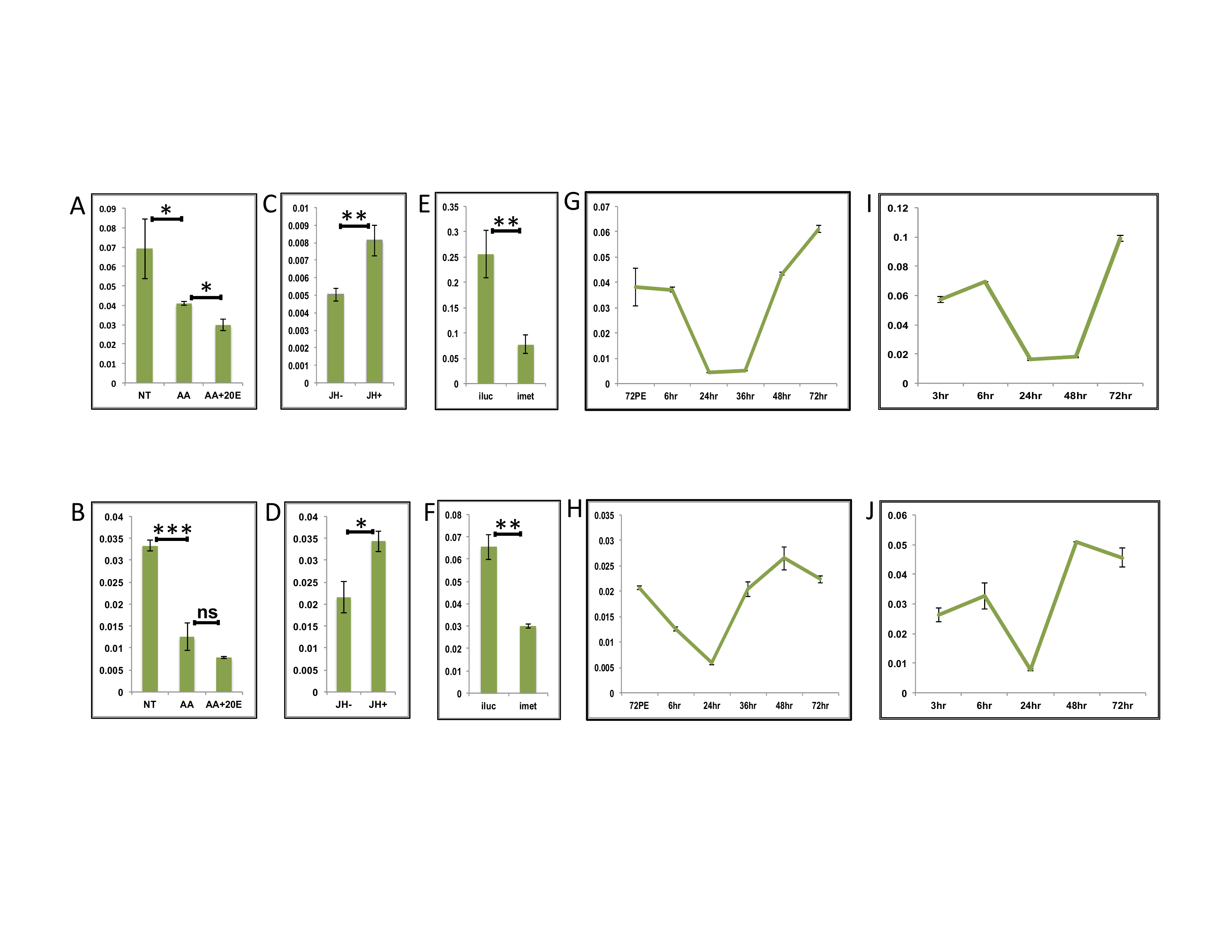

Supplement: S9 Fig — (A-B) Relative expression of genes—AAEL002675, arginase and AAEL001623, proteasome subunit, detected by qRT-PCR, in tissues subjected to in-vitro fat body culture (IVFBC) in culture media without (NT) and with amino acids (AA) and with amino acid plus 20E (AA+20E) (C-D) Relative expression of the same genes in tissues subjected to IVFBC in culture media without (JH-) and with (JH+) juvenile hormone. (E-F) Relative expression of the same genes detected by qRT-PCR, in fat body tissues collected from female mosquitoes post Met knock-down (iMet). Injecting double stranded RNA for the Luciferase gene (iluc) served as the control in the RNAi experiments. (G-H) Expression profiles of the same genes post first blood meal. (I-J) Expression profiles of the genes after the completion of the first reproductive cycle (egg laying) and post second blood meal. All expression calculated against housekeeping gene RPS7. Data representative of three biological replicates, with three technical replicates and are illustrated as average ± SD, * P < 0.05; ** P < 0.01; *** P < 0.001. (TIFF) [file pgen.1005450.s009.tiff]

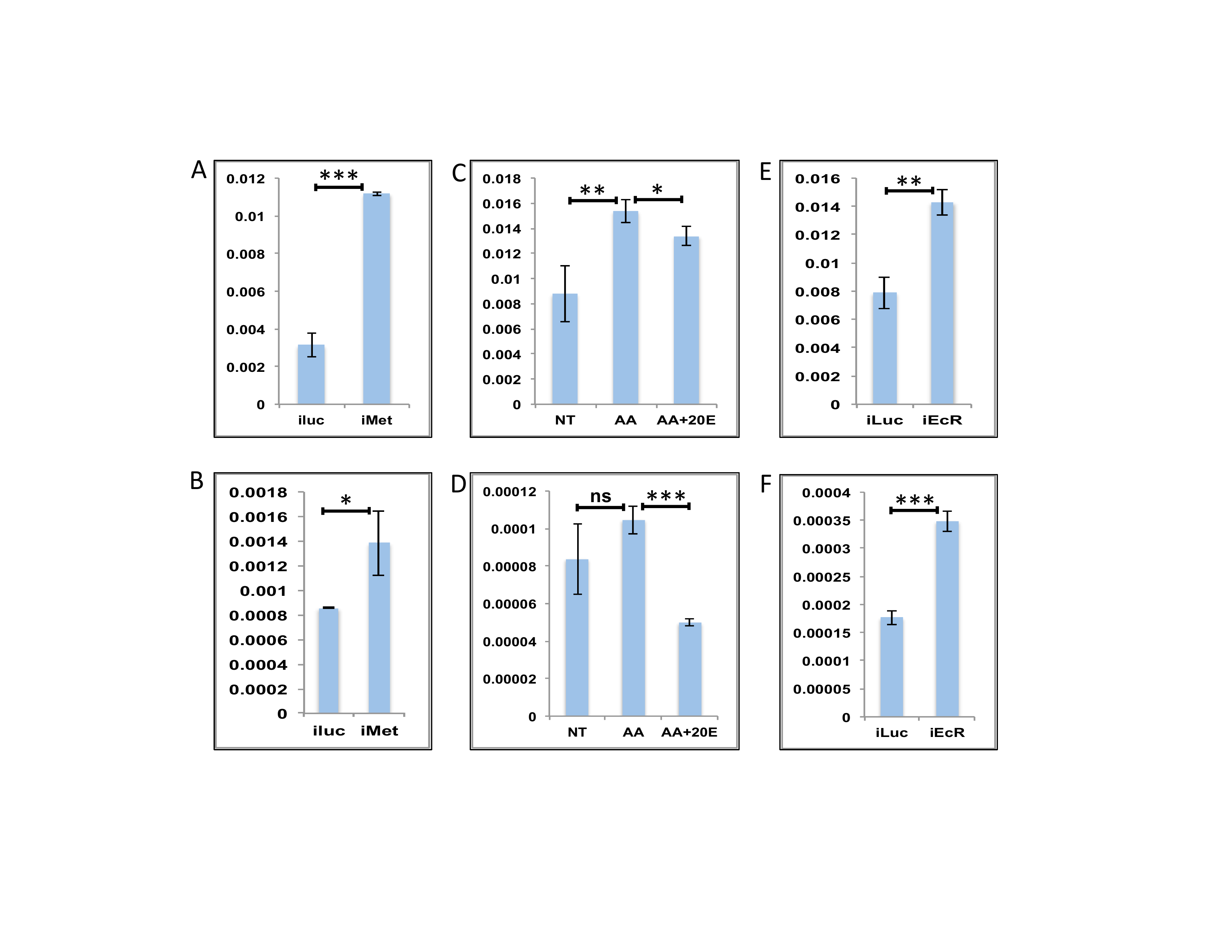

Supplement: S10 Fig — (A-B) Relative expression of the genes—AAEL003347, CRAL/TRIO domain containing protein and AAEL000705, Steroid dehydrogenase, detected by qRT-PCR, in fat body tissues collected from female mosquitoes post Met knock-down (iMet); injecting double stranded RNA for the Luciferase gene (iluc) served as the control. (C-D) Relative expression of the same genes detected by qRT-PCR, in tissues subjected to in-vitro fat body culture (IVFBC) in culture media without (NT) and with amino acids (AA) and with amino acid plus 20E (AA+20E). (E-F) Relative expression of the same genes detected by qRT-PCR, in fat body tissues collected from female mosquitoes post EcR knock-down (iEcR) injecting double stranded RNA for the Luciferase gene (iluc) served as the control. (TIFF) [file pgen.1005450.s010.tiff]

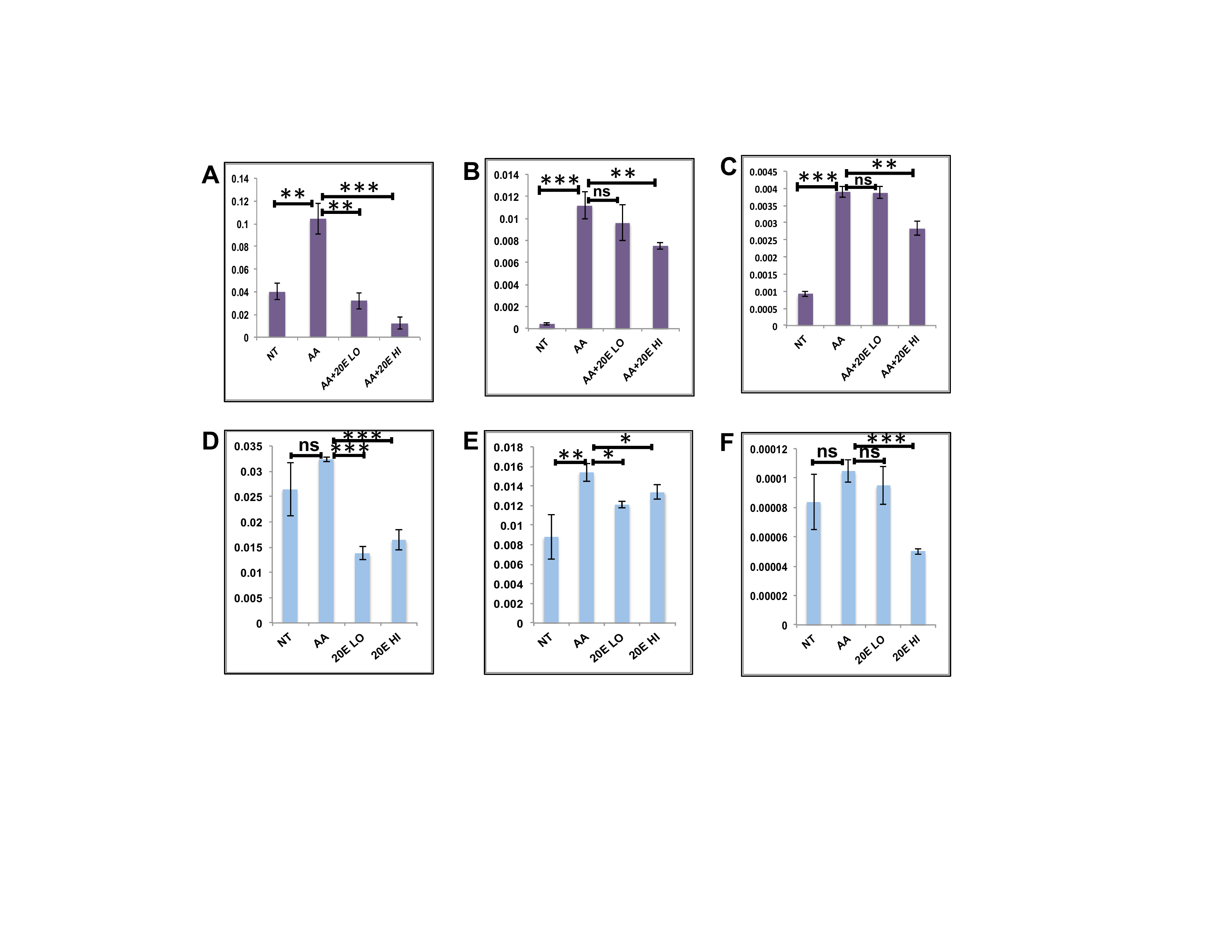

Supplement: S11 Fig — (A-C) Relative expression of the early genes—AAEL002269, Purine nucleoside phosphorylase (A), AAEL002488, Dead box atp dependent RNA helicase (B) and AAEL004345, Cysteinyl t-RNA synthetase (C) detected by qRT-PCR, in tissues subjected to in-vitro fat body culture (IVFBC) in culture media without (NT) and with amino acids (AA), with amino acid plus low concentration of 20E (AA+20E LO) and with amino acid plus high concentration of 20E (AA+20E HI). (D-F) Relative expression of the late-mid genes—AAEL002781, Galactokinase (D), AAEL003347, CRAL/TRIO domain containing protein (E) and AAEL000705, Steroid dehydrogenase (F) detected by qRT-PCR, in tissues subjected to in-vitro fat body culture (IVFBC) in culture media without (NT) and with amino acids (AA), with amino acid plus low concentration of 20E (AA+20E LO) and with amino acid plus high concentration of 20E (AA+20E HI). (TIFF) [file pgen.1005450.s011.tiff]

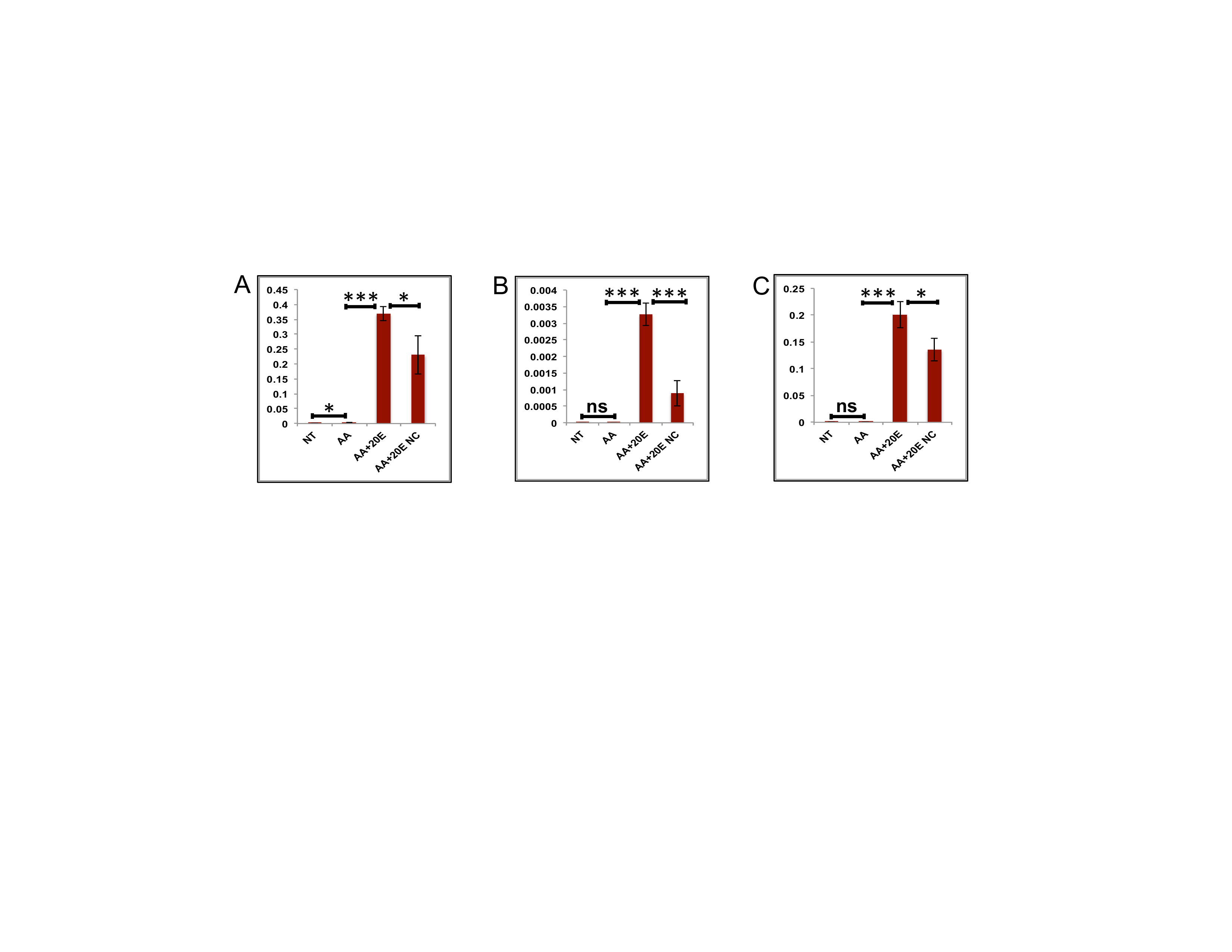

Supplement: S12 Fig — Genes respond significantly better when incubated with two different concentrations (5x 10^−8^ M and 10−6 M) of 20E, along with AAs (AA+20E) for 8 h (4 h + 4 h respectively), in IVBFC, than, when incubated for 8 h with the higher concentration of 20E plus AAs (AA+20E NC). The YPP genes–(A) Vitellogenin, (B) Cathepsin beta and (C) Carboxypeptidase were used for this test. Data representative of three biological replicates, with three technical replicates and are illustrated as average ± SD, * P < 0.05; ** P < 0.01; *** P < 0.001. (TIF) [file pgen.1005450.s012.tif]
