# Supplementary material for: Regulation of Gene Expression Patterns in Mosquito Reproduction
Source: PLoS Genet. 2015 Aug 14;11(8):e1005450. doi: 10.1371/journal.pgen.1005450 (PMC4537244; doi:10.1371/journal.pgen.1005450)
Supplement: S1 Reference — (DOCX) [file pgen.1005450.s018.docx]

1. Hagedorn HH, Shapiro JP, Hanaoka K (1979) Ovarian ecdysone secretion is controlled by a brain hormone in an adult mosquito. *Nature.* 282(5734):92-94
